# Overexpression of Catalase Diminishes Oxidative Cysteine Modifications of Cardiac Proteins

**DOI:** 10.1371/journal.pone.0144025

**Published:** 2015-12-07

**Authors:** Chunxiang Yao, Jessica B. Behring, Di Shao, Aaron L. Sverdlov, Stephen A. Whelan, Aly Elezaby, Xiaoyan Yin, Deborah A. Siwik, Francesca Seta, Catherine E. Costello, Richard A. Cohen, Reiko Matsui, Wilson S. Colucci, Mark E. McComb, Markus M. Bachschmid

**Affiliations:** 1 Vascular Biology Section, Department of Medicine, Whitaker Cardiovascular Institute, Boston University School of Medicine, Boston, Massachusetts, United States of America; 2 Myocardial Biology Unit, Department of Medicine, Whitaker Cardiovascular Institute, Boston University School of Medicine, Boston, Massachusetts, United States of America; 3 Cardiovascular Proteomics Center, Center for Biomedical Mass Spectrometry, Boston University School of Medicine, Boston, Massachusetts, United States of America; 4 Boston University and National Heart, Lung and Blood Institute’s Framingham Heart Study, Framingham, Massachusetts, United States of America; 5 Department of Biostatistics, Boston University School of Public Health, Boston, Massachusetts, United States of America; University of Nebraska-Lincoln, UNITED STATES

## Abstract

Reactive protein cysteine thiolates are instrumental in redox regulation. Oxidants, such as hydrogen peroxide (H_2_O_2_), react with thiolates to form oxidative post-translational modifications, enabling physiological redox signaling. Cardiac disease and aging are associated with oxidative stress which can impair redox signaling by altering essential cysteine thiolates. We previously found that cardiac-specific overexpression of catalase (Cat), an enzyme that detoxifies excess H_2_O_2_, protected from oxidative stress and delayed cardiac aging in mice. Using redox proteomics and systems biology, we sought to identify the cysteines that could play a key role in cardiac disease and aging. With a ‘Tandem Mass Tag’ (TMT) labeling strategy and mass spectrometry, we investigated differential reversible cysteine oxidation in the cardiac proteome of wild type and Cat transgenic (Tg) mice. Reversible cysteine oxidation was measured as thiol occupancy, the ratio of total available versus reversibly oxidized cysteine thiols. Catalase overexpression globally decreased thiol occupancy by ≥1.3 fold in 82 proteins, including numerous mitochondrial and contractile proteins. Systems biology analysis assigned the majority of proteins with differentially modified thiols in Cat Tg mice to pathways of aging and cardiac disease, including cellular stress response, proteostasis, and apoptosis. In addition, Cat Tg mice exhibited diminished protein glutathione adducts and decreased H_2_O_2_ production from mitochondrial complex I and II, suggesting improved function of cardiac mitochondria. In conclusion, our data suggest that catalase may alleviate cardiac disease and aging by moderating global protein cysteine thiol oxidation.

## Introduction

Excessive oxidant formation is associated with cardiac disease and aging [1]. In an attempt to ameliorate cardiac diseases and delay aging by decreasing oxidative stress, mouse models overexpressing cellular antioxidant enzymes, including catalase, were created. Catalase detoxifies the intracellular oxidant hydrogen peroxide (H_2_O_2_) [2]. We and others have previously shown that cardiac-specific catalase overexpression in transgenic mice (Cat Tg) prevents oxidative stress, preserves diastolic heart function and delays cardiac aging [3–5]. In addition, Cat Tg mice are also protected from doxorubicin toxicity [6], ischemia-reperfusion injury [7,8], inflammation [9], and overt heart failure [10].

H_2_O_2_ can have dual effects; acting at physiological levels as an essential signaling molecule or in excess as a damaging oxidant [11]. Protein cysteine thiolates (Cys-S^-^), as compared to thiols (Cys-SH), are chemically reactive and confer redox sensitivity. These thiolates or “redox sensors” can react with H_2_O_2_ to form a labile sulfenic acid (S-OH) intermediate, which quickly reacts with the abundant intracellular low-mass thiol glutathione (GSH). The resulting protein GSH-adduct is a reversible oxidative protein modification that participates in cellular redox signaling and regulates protein activity, interaction, localization, and stability [11,12]. Redox signaling, however, becomes dysregulated or impaired when oxidants are generated in excess, and this leads to oxidative stress and irreversible modifications as observed in cardiac disease and aging.

We studied reversible protein cysteine thiol oxidation in heart lysates from adult wild type and Cat Tg mice. Iodoacetyl ‘Tandem Mass Tags’ (iodoTMT) developed for multiplexed quantitative mass spectrometry were used in an approach similar to established biotin-switch assays to label and analyze oxidation of protein cysteine thiols [13]. Using this labeling strategy in combination with proteomics mass spectrometry, we determined the ratio of reversibly oxidized cysteine thiols to total available cysteines—referred to as thiol occupancy—on a site-specific basis for wild type and Cat Tg mice. Significant differences in thiol occupancy for Cat Tg and WT mice identify the most reactive and, therefore, potential functionally relevant cysteines in proteins. We aimed to provide evidence that by overexpressing catalase, reactive cysteine thiols are maintained in a functional state and protected from the irreversible oxidation observed in cardiac disease and aging. Our results provide novel insights into catalase function that, beyond ameliorating oxidative stress, may also influence protective cellular signaling pathways.

## Materials and Methods

### Experimental animals and materials

Male and female FVB/N mice (N = 5 per group) with cardiac-specific catalase overexpression and wild type controls were used. The protocol was approved by the Institutional Animal Care and Use Committee at Boston University School of Medicine. Mice were euthanized at 10 months of age and hearts were perfused to remove blood proteins, excised, snap frozen, and stored in liquid nitrogen or at -80°C. All materials were purchased from Thermo Fisher Scientific (Waltham, MA, USA) unless otherwise specified.

### Mitochondrial isolation

Heart mitochondria were isolated as we have described previously, with minor modifications [14,15]. All steps were performed on ice. Briefly, tissues were rinsed in a buffer containing 100 mM KCl, 5 mM EGTA and 5 mM HEPES pH 7.4, and thereafter homogenized in 2 ml of HES buffer (HEPES 5 mM, EDTA 1 mM, sucrose 0.25 M, pH 7.4 adjusted with KOH 1 M) using a Teflon-on-glass electric homogenizer. The homogenate was centrifuged at 500 × *g* for 10 min at 4°C. The supernatant was then centrifuged at 9000 × *g* for 15 min at 4°C and the mitochondrial pellet was re-suspended in 100 μl of HES buffer with 0.3% of fatty acid-free bovine serum albumin. Protein was quantified using the BCA assay (Pierce) and the value of HES-BSA buffer alone was subtracted.

### H_2_O_2_ production in isolated mitochondria

Mitochondrial H_2_O_2_ production in isolated cardiac mitochondria was measured using the Amplex Ultra Red-Horseradish peroxidase method (Invitrogen) as we described previously, with minor modifications [15]. This assay is based on the Horseradish peroxidase (2 units/ml) H_2_O_2_-dependent oxidation of non-fluorescent Amplex Ultra Red (50 μM) to fluorescent resorufin red. In short, 10 μg mitochondria were diluted in 50 μl reaction buffer (125 mM KCl, 10 mM HEPES, 5 mM MgCl_2_, 2 mM K_2_HPO_4_, pH 7.44) to determine complex I- (pyruvate/ malate, 5 mM) or complex II- (succinate, 5 mM; with and without inhibitor, rotenone 2 μM) driven H_2_O_2_ production. Mitochondrial H_2_O_2_ production was measured after the addition of 50 μl of reaction buffer containing horseradish peroxidase and Amplex Ultra Red. Fluorescence was followed at an excitation wavelength of 545 nm and an emission wavelength of 590 nm for 20 min. The slope of the increase in fluorescence is converted to the rate of H_2_O_2_ production with a standard curve. All of the assays were performed at 25°C. The results are reported as pmoles/min/mg protein.

### ATP production in isolated mitochondria

ATP synthesis rates in isolated heart mitochondria were determined using the luciferin/luciferase based ATP Bioluminescence Assay Kit CLS II (Roche) as we have previously described, with minor modifications [15]. In short, 10 μg of heart mitochondria were suspended in 75 μl buffer A (125 mM KCl, 10 mM HEPES, 5 mM MgCl_2_ and 2 mM K_2_HPO_4_, pH7.44) to determine complex I- (pyruvate/ malate, 5 mM final) or complex II- (succinate, 5 mM final) driven ATP synthesis. Following standard practice, succinate-driven ATP generation was measured in the presence of complex I inhibitor rotenone (2 μM) to avoid the reverse electron transfer effect [16]. Measurements with substrates were repeated in the presence of oligomycin to determine the rates of non-mitochondrial ATP production. The background of the assay was determined with mitochondria alone. The measurements for all samples were started simultaneously by adding 75 μl of luciferin/luciferase buffer containing 1 mM ADP (0.5 mM final). The initial slope of the increase in ATP-supported luciferase chemiluminescence was used to determine the rate of ATP production after subtraction of the background and non-mitochondrial values. Using an ATP standard provided in the kit, the slopes were converted to nmoles/min/mg protein.

### Oxygen consumption rate in isolated mitochondria

Oxygen consumption rates were monitored using a Seahorse XF24 oxygen flux analyzer, as previously described by our group [14]. Isolated mitochondria were loaded in a 24-well Seahorse plate on ice (5–12.5 μg per well) and 500 μl of ice-cold mitochondrial assay solution (MAS: 70 mM sucrose, 220 mM mannitol, 5 mM KH_2_PO_4_, 5 mM MgCl_2_, 2 mM HEPES, 1 mM EGTA, 0.3% BSA fatty acid-free, pH 7.4) were added on top. The 4 sequential injection ports of the Seahorse cartridge contained the following: Port A- 50 μl of 10X substrate (complex I: 50 mM pyruvate and 50 mM malate; complex II: 50 mM succinate and 20 μM rotenone in MAS) and 2.5 mM ADP, Port B- 55 μl of 20 μM oligomycin, Port C- 60 μl of 40 μM FCCP, Port D- 65 μl of 40 μM antimycin A. State III was determined after port A injection, state IV after port B, and uncoupled after port C. Antimycin A was used as a control because it blocks the electron transport chain to minimize mitochondrial oxygen consumption. The results are reported as ρmol oxygen/min/μg protein.

### Immunoblotting

Hearts were lysed in RIPA buffer (50mM Tis-HCl, pH 7.2, 0.15 M NaCl, 1.0 mM EDTA, 0.1% SDS, 1.0% Triton X-100, 1.0% sodium deoxycholate). Tissue lysates were separated on non-reducing SDS-polyacrylamide gels and transferred to polyvinylidene fluoride membranes (Immobilon, Millipore), blocked in 5% nonfat milk, and incubated overnight with the anti-GSH antibody (Virogen, 101-A). Subsequently, blots were incubated with IR dye-conjugated secondary antibodies and visualized using the LI-COR Odyssey system. Band intensities were measured using ImageJ, and values were normalized to GAPDH as a loading control.

### Homogenization and protein extraction from heart tissue for mass spectrometry analysis

The left heart ventricle was divided into two pieces; homogenization and extraction of individual pieces were carried out in lysis buffer containing 50 mM Tris pH 7.4, 150 mM NaCl 0.5 mM diethylene triamine pentaacetic acid (DTPA) (Sigma, St. Louis, MO) and protease inhibitor cocktail (Roche Applied Science, Indianapolis, IN) (1 tablet/10 ml of lysis buffer) supplemented with either 10 mM iodoacetamide (Sigma) to block free thiols or 1 mM tris(2-carboxyethyl)phosphine (TCEP, Thermo Fisher Scientific) to completely reduce all reducible thiols. LVs were homogenized for two cycles of 54 sec in a gentleMACS Dissociator (MACS Miltenyl Biotec, Cologne, Germany). Mass spectrometry compatible RapiGest SF surfactant (0.1%, v/v, Waters, Milford, MA) was subsequently introduced into the lysis mixture to avoid foaming during homogenization. Lysates were rotated for 2 h at 4°C, aliquotted, and frozen at -80°C. The protein concentration for each sample was determined using the colorimetric Bio-Rad detergent-compatible protein assay (BioRad, Hercules, CA) and by Imperial staining (Thermo Fisher Scientific) of sodium dodecyl sulfate-polyacrylamide gels (SDS-PAGE).

### iodoTMT-labelling and enrichment

One hundred μg of each protein lysate was used to label the reversibly oxidized thiols or total available (all reducible) thiols, from either control or Cat Tg LV homogenates. Then 7-kDa MWCO Zeba spin columns (Pierce, Thermo Scientific) equilibrated with Tris-buffered saline (TBS: 25mM Tris and 0.15 M NaCl, pH 7.2–7.5) containing 5% acetonitrile (ACN) and 0.005% ProteaseMAX (Promega, Madison, WI) were used to remove excess IAM from blocked samples before 45 min of reduction with TCEP at 37 ^o^C. Completely reduced samples were thawed and passed through 7kDa MWCO Zeba columns pretreated with TCEP-containing buffer, and incubated to maintain conditions similar to blocked samples. Next, each sample was tagged with a different isobaric iodoacetyl Tandem Mass Tag™ (iodoTMT™) reagent (Thermo Scientific) at 1 mM for 2 h at room temperature. Samples representing all four conditions were combined, then precipitated with acetone containing 0.1% acetic acid at -20°C overnight. Protein pellets were washed with ice-cold acetone, and then resuspended in 1x Tris supplemented with 0.05% ProteaseMAX and 5% ACN. Proteins were digested with trypsin (1:20 enzyme-to-protein ratio) overnight at 37°C. Immobilized anti-TMT resin was used to immunoprecipitate (IP) TMT-tagged peptides at a 1 μg protein/1 μl resin ratio overnight at 4°C. Resin was washed three times with TBS, three times with 4 M urea, and four times with water. The TMT-tagged peptides were eluted with TMT-elution buffer (Thermo Scientific) and concentrated in a vacuum concentrator at 4°C. Peptides were dissolved in 0.1% formic acid (FA) in 2% ACN and desalted with C18 spin columns (NestGroup). Peptides were eluted from the C18 columns twice with 0.1% FA in 60% ACN. Samples were then dried using a SpeedVac™ concentrator (Savant, Thermo Fisher Scientific) and reconstituted in 2% ACN with 0.1% FA. Preliminary MS analysis was performed with an UltrafleXtreme MALDI-TOF/TOF MS (Bruker Daltonics, Billerica, MA) using 2,5-dihydroxybensoic acid matrix, followed by analysis with the Q Exactive™ mass spectrometer described below.

### Liquid chromatography-tandem mass spectrometry (LC-MS/MS)

LC-MS/MS was performed on peptide samples using a nanoAcquity UPLC™ nano-capillary high-performance LC system (Waters Corp., Milford, MA) coupled to a Q Exactive™ hybrid quadrupole-Orbitrap mass spectrometer (Thermo Fisher Scientific, San Jose, CA) equipped with a TriVersa NanoMate ion source (Advion, Ithaca, NY). Sample concentration and desalting were performed online using a nanoAcquity UPLC™ trapping column (180 μm x 20 mm, packed with 5 μm, 100 Å Symmetry C18 material, Waters Corp.) at a flow rate of 15 μl/min for 1 min. Separation was accomplished on a nanoAcquity UPLC™ capillary column (150 μm x 100 mm, packed with 1.7 μm, 130 Å BEH C18 material, Waters Corp.). A linear gradient of A and B buffers (buffer A: 1.5% ACN/ 0.1% FA; buffer B: 98.5% ACN/ 0.1% FA) from 3% to 55% buffer B over 120 min was used at a flow rate of 0.5 μl/min to elute peptides into the mass spectrometer. Columns were washed and re-equilibrated between LC-MS/MS experiments. Electrospray ionization was carried out at 1.65 kV using the NanoMate, with the Q Exactive heated transfer capillary set to 250°C. Mass spectra were acquired in the positive-ion mode over the range *m/z* 400–2000 at a resolution of 70,000 (fwhm at *m/z* 400), target AGC values of 1E6, and maximum individual fill times of 200 ms. Mass accuracy after internal calibration was within 1 ppm. MS/MS spectra were acquired at a resolution of 17,500 (fwhm at *m/z* 100), target AGC values of 1E6, and isolation window of 3.0 *m/z*, using higher-energy collisional dissociation (HCD) with 30% MS/MS collision energies (NCE) and nitrogen as the collision gas. Maximum individual fill time was set as 60 ms for the 10 most abundant, multiply-charged species in each MS spectrum with signal intensities of > 3.3E5. MS/MS spectra were acquired over an *m/z* range that depended on each precursor ion. Dynamic exclusion was set such that MS/MS for each species was excluded for 2 sec post-acquisition. All spectra were recorded in profile mode for further processing and analysis.

### Data analysis

MS and MS/MS data analyses were carried out using Proteome Discoverer (PD) 1.4 (Thermo Fisher Scientific). For Protein/peptide identification, the MS/MS spectra were searched against the UniProtKB mouse protein database (20130107/83589 sequence entries) using the Sequest HT search engine. The search was conducted with the following constraints: full tryptic peptides with maximum four missed cleavage sites, precursor mass tolerance 10 ppm, and fragment ion mass error 0.02 Da. IodoTMT6plex-labeled (C), di- and tri-oxidized (C), carbamidomethyl (C), deamidation (NQ), and oxidation (M) were specified as dynamic modifications. False discovery rate (FDR) was determined through decoy database search using Percolator node for peptide-spectrum-match (PSM) validation. Peptides assignment with FDR<1% were indicated as highly confident and selected for further analysis.

For relative quantification of TMT-labeled cysteines, reporter ions were specified with the integration tolerance of 20 ppm using most confident centroid method. Thermo lot-specific reporter ion correction factors were applied for “quan value” determination. Protein GO annotations and subcellular locations were derived from open source software STRAP (Cardiovascular Proteomics Center, Boston University School of Medicine, Boston, MA) [17,18]. Peptide events without iodoTMT modification or without matched protein were excluded. For each MS/MS spectrum used for quantification, PD used the intensity of each reporter ion to calculate the following ratios between biological samples: 1) changes in total available cysteines and 2) changes in reversibly oxidized cysteine thiols. Only the median ratio was reported as the change for the assigned peptide. Thiol occupancy caused by reversible oxidation was calculated as the ratio of reversibly oxidized thiol to the total available thiols for the same peptide. These values were then compared between mice. TMT-tagged peptides having less than 35% coefficients of variation (CVs) among measured replicate mice, both for their change in total available cysteine and reversibly oxidized cysteines, were selected for further analysis. Fold changes between samples were expressed as log2 value of the ratios for all quantifiable peptides. The proteins exhibiting changes in reversible thiol oxidation (cutoff ≥1.3-fold change is common threshold for gene chip and RT-qPCR analysis) were analyzed for functional relevance using Ingenuity™ Pathway Analysis (IPA) (IPA™, QIAGEN Redwood City, www.qiagen.com/ingenuity). Direct and indirect relationship data derived from all wild type and mutant cells and tissue from mouse, rat, and human were included. Endogenous chemicals were also included in the canonical pathway analysis.

## Results and Discussion

### Detection of reversible cysteine oxidation with TMT switch and mass spectrometry

**Fig 1:**
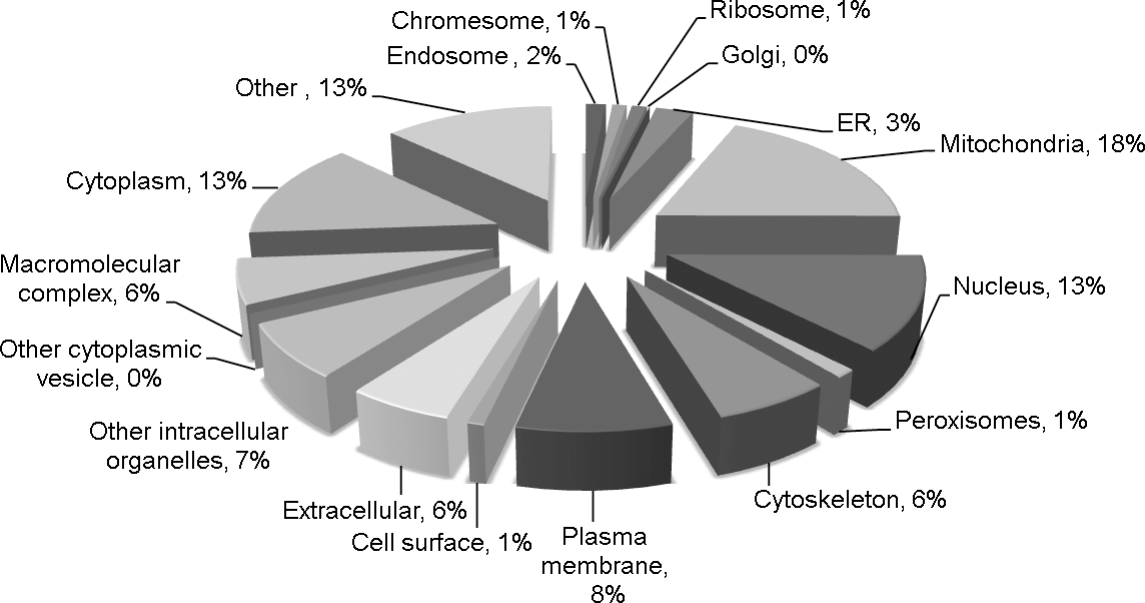
Detection of reversible cysteine thiol oxidation with TMT switch and mass spectrometry. Cellular compartment pie chart of 1017 proteins containing TMT-tagged cysteine thiols.

Using our previously published iodoTMT switch assay and mass spectrometry [13] (S1 Fig), reversible protein cysteine thiol oxidation and total available cysteines were measured in left ventricles from wild type and Cat Tg mice (N = 5). In summary, 2125 TMT-tagged peptides were identified (S1 Table), which originated from 1017 proteins localizing to various subcellular compartments, including mitochondria (18%), cytoplasm (13%), nucleus (13%), plasma membrane (8%), cytoskeleton (6%) and peroxisome/microbodies (1%) (Fig 1).

### Global changes in protein cysteine thiol oxidation associated with catalase overexpression

**Fig 2:**
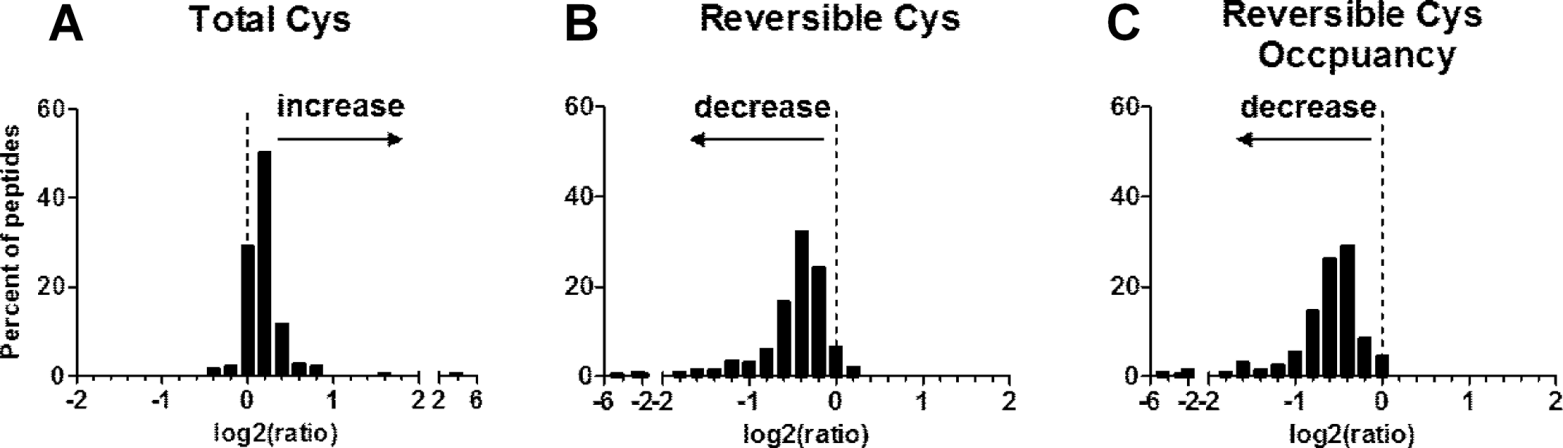
Global changes in cysteine oxidation associated with catalase overexpression. Log2 transformed reporter ion rations from Cat Tg *vs*. WT mice were used to generate frequency histograms of TMT-tagged peptides. The ratios used are as follows: (A) changes in the total available cysteines; (B) changes in the reversibly oxidized cysteine thiols; (C) changes in the thiol occupancy of reversible oxidation (dashed line indicates no change with log2 ratio = 0).

The global effects of catalase overexpression on total available (Fig 2A) and reversibly oxidized cysteine thiols (Fig 2B), as well as thiol occupancy (Fig 2C) was measured for 199 qualifying peptides (S2 Fig) from 147 proteins, which are depicted in a log2 transformed frequency histogram. Reversible cysteine thiol oxidation was significantly decreased in Cat Tg mice (a log2 transformed mean of -0.52 indicates a 1.4-fold decrease in Cat Tg *vs*. WT, Fig 2B). Calculating the ratio of reversibly oxidized to total available cysteine thiols enables quantification of changes in thiol occupancy, and can be used to differentiate regulatory cysteines in adult Cat Tg mice. The thiol occupancy (Fig 2C, a log2 transformed mean of -0.68 indicates a 1.6-fold decrease) was significantly decreased in Cat Tg mice and paralleled that of reversible cysteine thiol oxidation.

Because no experimental precautions were taken to preserve very labile and transient cysteine modifications, including *S*-nitrosylation and *S*-sulfenylation [19,20], and owing to high intracellular glutathione levels [21], we deem that most of the thiol modifications are either protein GSH adducts or disulfides. However, the type of modification needs to be determined in future studies, on a case-by-case basis.

Overall, 17 proteins with increased total available cysteine thiols, and 82 proteins with decreased reversible cysteine thiol oxidation exhibited biologically significant changes in Cat Tg *vs*. WT mice. These proteins are involved in various cellular pathways that protect from oxidative and metabolic stress, protein misfolding and apoptosis. Proteins with a ≥2-fold change in thiol occupancy are summarized in S2 Table. Below, we speculate about and discuss the potential functional significance of thiol modifications of some of these proteins in the context of cardiac disease and aging.

### Effects of overexpression on catalase itself and other antioxidant systems

We measured a 14-fold increase in total available cysteines for catalase (Cat, Q3UF58) from Cat Tg mice, clearly indicating catalase overexpression and thus, validating our proteomics approach. Moreover, the regulatory site of catalase activity (Cys376) [22], exhibited a 24.5-fold decrease in thiol occupancy.

Catalase is highly efficient in detoxifying large quantities of H_2_O_2_ and preventing oxidative stress. These properties could protect other cellular antioxidants from oxidation and improve their effectiveness. Peroxiredoxins, which also decompose H_2_O_2_, are highly redox-sensitive proteins susceptible to irreversible oxidation [23,24]. Cys96, one of the two redox-active cysteines in peroxiredoxin 5, showed decreased cysteine thiol oxidation, which likely improves its activity (Prdx5, Q9JHL8, 1.2-fold decrease in thiol occupancy in Cat Tg *vs*. WT).

The redox-sensitive substrate binding sites of carbonyl reductase 1, an NADPH-dependent short chain dehydrogenase/reductase [25–27], also showed attenuated oxidation in Cat Tg mice (Cbr1, P48758, 3.0-fold decrease in thiol occupancy of Cys226 and Cys227 in Cat Tg *vs*. WT). Cbr1 has been linked to the detoxification of reactive aldehydes, such as the lipid peroxidation product 4-hydroxynonenal, under conditions of oxidative stress. Moreover, Cbr1 regulates intracellular nitrosoglutathione levels [28], an intracellular storage form of nitric oxide, which would impact cardiovascular physiology if the observed decrease in oxidation protects Cbr1 function.

### Mitochondria

Dysfunctional mitochondria are now recognized as a factor contributing to disease and aging [1,29]. Catalase overexpression improved mitochondrial function in adult mice and attenuated cysteine thiol oxidation of various proteins involved in apoptosis and mitochondrial metabolism.

Pyruvate dehydrogenase (Pdha1, P35486) is a mitochondrial matrix enzyme that catalyzes oxidative decarboxylation of pyruvate, producing acetyl-CoA that directly feeds into the citric acid cycle. The enzyme plays a fundamental role in cardiac metabolism and adaptation to hypoxic conditions [30]. Our proteomics study identified three cysteine sites with decreases in thiol occupancy (1.3-fold for Cys181; 6.8-fold for Cys218 and Cys222 in Cat Tg *vs*. WT), which would fit with previous reports that found these sites can be S-nitrosylated in the myocardium [31].

Mammalian multiple mitochondrial dysfunctions syndrome 2 protein (MMDS2; also BolA3, E9Q705) belongs to the BolA-like protein family that has been postulated to act as a reductase for mitochondrial glutaredoxin [32,33]. BolA3 is involved in the assembly of iron-sulfur cluster-containing mitochondrial proteins [33,34] and lipoate synthesis, directly affecting the function of pyruvate dehydrogenase. Fibroblasts deficient in BolA3 exhibit perturbations in the citric acid cycle and mitochondrial electron transport chain. Thus BolA3 would improve mitochondrial respiration and ATP synthesis in Cat Tg mice if the observed decrease in thiol oxidation increases BolA3 activity (Cys47, 2.9-fold decrease in thiol occupancy, Cat Tg *vs*. WT). In our recent publications [13,15], increased cysteine oxidation in iron-sulfur clusters was associated with metabolic heart disease. Particularly, cysteines of the complex II iron-sulfur cluster were oxidized [15], which may contribute to increased oxidant formation in cardiac and age-related diseases.

Mitochondrial protein serine hydroxymethyltransferase 2 (SHMT2, Q3TFD0, Cys77, 1.4-fold decrease in thiol occupancy, Cat Tg *vs*. WT) is involved in ‘one-carbon’ and folate metabolism [35]. These metabolites are essential for nucleotide biosynthesis, numerous methylation reactions and maintaining low homocysteine levels. Increased serum homocysteine is a well-established cardiovascular risk factor and would promote endothelial dysfunction and atherosclerosis if cysteine oxidation leads to enzymatic inhibition of SHMT2.

Voltage-dependent anion-selective channel proteins (VDAC) [36,37] facilitate passive exchange of ions and transport of ATP, ADP, pyruvate, malate, and other metabolites [38,39]. In conjunction with proteins of the Bcl-2 family, VDACs form the permeability transition pore. During oxidant-mediated apoptosis, the pore opens, facilitating the release of cytochrome C and initiation of additional apoptotic cascades [40,41]. For VDAC1, two cysteines—Cys127 and Cys232—are essential for crosslinking high order oligomers upon apoptosis induction [42]. Because, Cys232 is proposed to play a particular role in apoptosis [42], a decrease in reversible cysteine oxidation could indicate suppression of myocyte apoptosis (VDAC1, Q3U6K8, Cys232, 2.1-fold decrease in thiol occupancy; VDAC2, G3UX26, 2.0-fold decreased in thiol occupancy for Cys36, 2.8-fold for Cys65, and 23.6-fold for in Cys199 and Cys216, Cat Tg *vs*. WT) [43]. Attenuating myocyte apoptosis by protecting key mediators from oxidation may preserve the functional integrity of the myocardium.

### Extracellular matrix and contractile proteins

Myocardial dysfunction may be caused by various factors including dysregulated calcium homeostasis [4] or cross-links of intracellular filamentous and extracellular matrix proteins. Both effects can lead to cardiac muscle stiffening and impairment of relaxation. Catalase overexpression attenuated cysteine thiol oxidation in various cardiac proteins associated with the contractile apparatus, *e*.*g*. titin isoform 2 (Ttn, A2ASS6-2, 2.3-fold decrease in thiol occupancy of Cys23752 in the fibronectin type-III domain, Cat Tg *vs*. WT); cardiac-type myosin-binding protein C (Mybpc3, Q3TF37, 2.5-fold of decrease in thiol occupancy Cys1040 and Cys1041 in the Ig-like C2-type domain, Cat Tg *vs*. WT), and the positive regulator of actin filament polymerization actin-related protein 2/3 complex subunit 2 (Arpc2, Q4FZG5, 1.7-fold decrease in thiol occupancy of Cys96 in Cat Tg *vs*. WT).

LIM domain-containing cysteine- and glycine-rich proteins (Csrp) are involved in development, cell growth, differentiation [44], and organization of cardiac contractile elements. The LIM domains serve as protein-binding interfaces to mediate subcellular protein localization and function [45]. For example, the first LIM domain and Gly-rich region in Csrp1 is necessary for bundling of actin filaments. Csrp3 is a positive regulator of myogenesis and plays a crucial role in the organization of the Z-line that also functions as a mechanical stretch sensor. In the hearts of Cat Tg mice, these proteins exhibited decreases in thiol occupancy. Thus cysteine oxidation of Csrp proteins may cause structural changes and defects in stretch sensing, leading to remodeling of the myocardium (Csrp1, P97315, 2-fold decrease in thiol occupancy for Cys 58; Csrp3, P50462, 3-fold decrease in thiol occupancy for Cys79 and 1.4-fold for Cys168, Cat Tg vs. WT).

The latent transforming growth factor ß–binding protein 4 (Ltbp4, Q8K4G1, 2.5-fold decrease in thiol occupancy of Cys1033, Cys1045 and Cys1051, Cat Tg *vs*. WT) is another factor that could be involved in cardiac remodeling. Ltbp4 belongs to the LTBP/fibrillin-family of extracellular matrix (ECM) proteins [46] and forms large latent complexes with transforming growth factor-ß (TGF-ß) in the ECM. These complexes regulate cell growth and differentiation, immune function, and ECM synthesis and degradation.

#### Effects of cysteine oxidation on protein networks

Pathway analysis (IPA, Ingenuity Systems) was performed with the 82 proteins exhibiting a decrease of ≥1.3-fold in reversible thiol oxidation in hearts of Cat Tg *vs*. WT mice. These proteins mainly participate in the “canonical pathways” of mitochondrial dysfunction, acute phase response signaling, TCA cycle, and NRF2-mediated oxidative stress response (S3 Table). Interestingly, most proteins with ≥2-fold decrease in reversible cysteine thiol oxidation were found in metabolic pathways of the mitochondria, suggesting that catalase overexpression may prevent or delay mitochondrial dysfunction in disease and aging. Prevention of damage and apoptosis could be essential to maintaining tissue homeostasis and function, particularly in post-mitotic tissues such as the myocardium which lack significant capacity for tissue regeneration.

**Fig 3:**
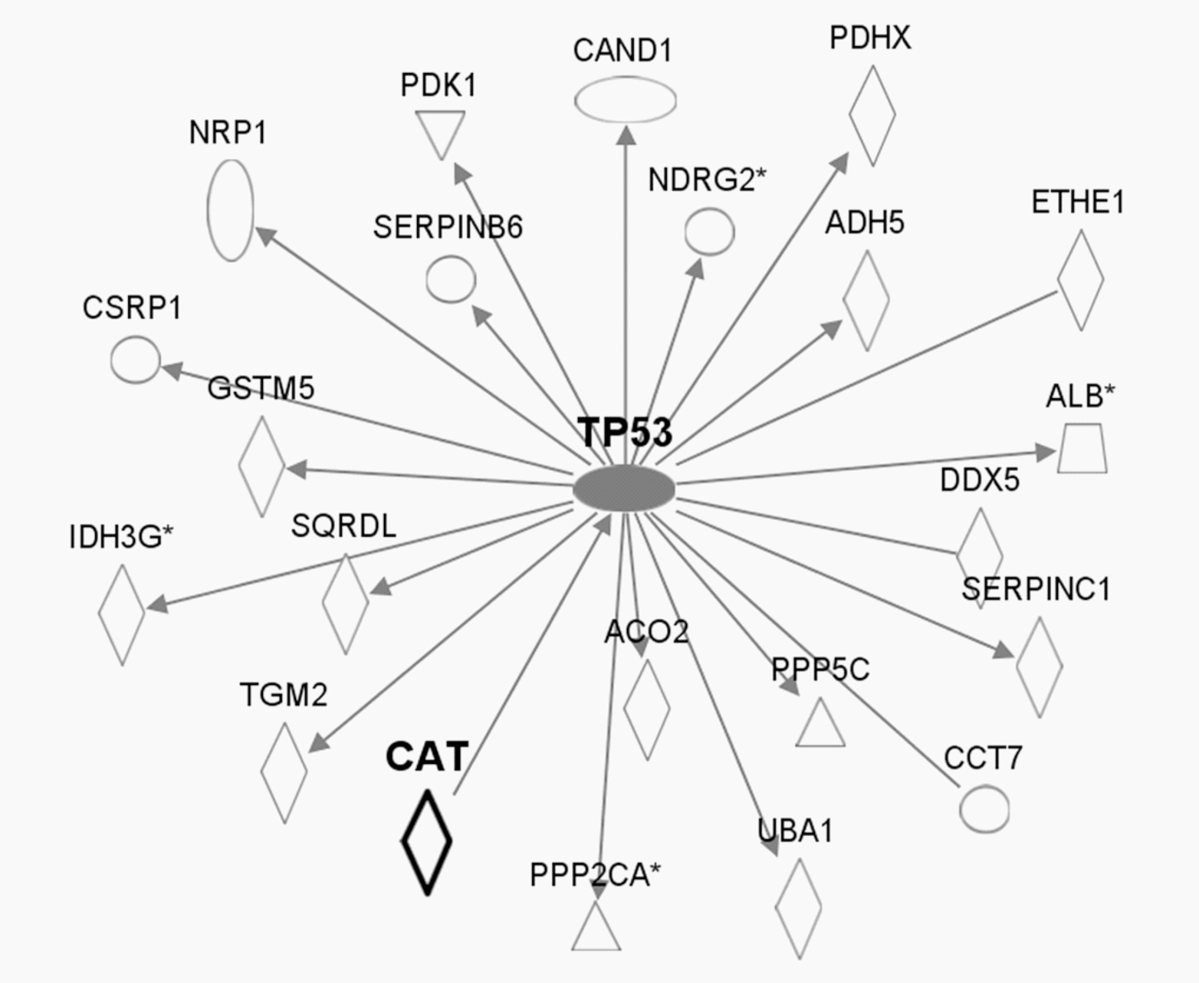
Pathway analysis-predicted protein networks associated with tumor suppressor p53. Legend to network analysis: enzyme (diamond), transmembrane receptor (vertical oval), transcriptional regulator (horizontal oval), phosphatase (triangle), transporter (trapezoid), kinase(triangle), growth factor (square), and other (circle). Relationships: interaction (line), activation (arrow).

**Fig 4:**
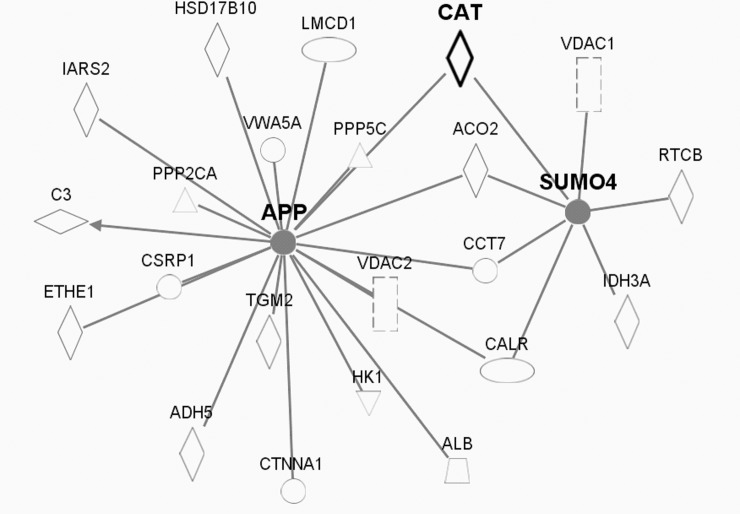
Pathway analysis-predicted protein networks associated with amyloid precursor protein (APP) and small ubiquitin-like modifier SUMO4. Legend to network analysis: enzyme (diamond), transmembrane receptor (vertical oval), transcriptional regulator (horizontal oval), phosphatase (triangle), transporter (trapezoid), kinase(triangle), growth factor (square), and other (circle). Relationships: interaction (—), activation (arrow).

**Fig 5:**
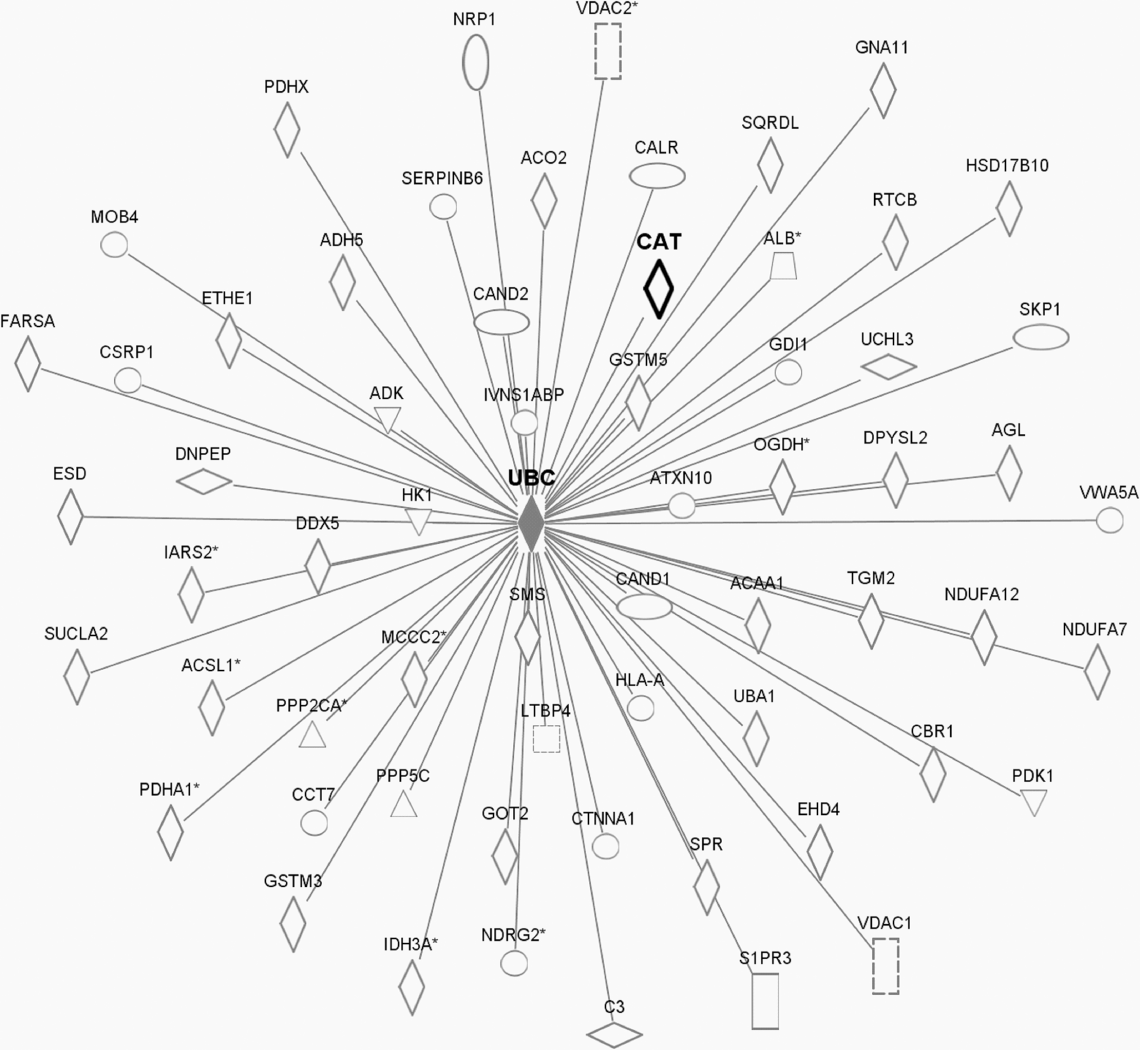
Pathway analysis-predicted protein networks associated with ubiquitin c (Ubc). Legend to network analysis: enzyme (diamond), transmembrane receptor (vertical oval), transcriptional regulator (horizontal oval), phosphatase (triangle), transporter (trapezoid), kinase(triangle), growth factor (square), and other (circle). Relationships: interaction (line), activation (arrow).

Our pathway analysis illustrates that 66 proteins with altered cysteine thiol oxidation in Cat Tg mice may be associated with catalase through 11 central protein ‘nodes’ (S3 Fig). Major ‘node’ proteins include the tumor suppressor p53 (Fig 3) and many involved in proteostasis i.e. amyloid precursor protein (APP), small ubiquitin-like modifier 4 (SUMO4) (Fig 4), and ubiquitin C (UbC) (Fig 5).

#### Tumor suppressor p53

The transcription factor and cell cycle regulator p53 is best known for its role as a tumor suppressor [47]. As a key component in the cellular stress response, p53 is activated by numerous signals, including genotoxic damage, oncogene activation, nutrient or oxygen deprivation, and oxidative stress [48]. Dampened stress response and attenuation of p53-dependent apoptosis probably leads to improved cell survival and could preserve tissue function [49]. In post-mitotic heart tissue, where tumor suppression is not a major concern, p53 likely perpetuates the disease and aging processes.

Pathway analysis indicated that p53 may function as a central node linking 21 proteins that exhibited differential thiol oxidation in Cat Tg mice (Fig 3). These proteins include regulatory subunits of serine/threonine-protein phosphatases that modulate oncogenic, insulin, and stress signaling cascades (Ppp2a, P63330, 2.0-fold decrease in thiol occupancy of Cys266; Ppp5c, F7BX26, 1.7-fold decrease in thiol occupancy of Cys76, Cat Tg vs. WT) [50,51]. Ppp2a targets various phosphorylation sites of p53, regulating its stability and transcriptional activity [52,53].

Another highly expressed p53-regulated cardiac protein with differential cysteine thiol oxidation is N-Myc downstream regulated gene 2 (Ndrg2, Q9QYG0-2, 1.7- and 1.4-fold decreased thiol occupancy of Cys64 and Cys241, respectively, Cat Tg vs. WT) [54–56]. Ndrg2 is a Myc-repressed gene that regulates cell growth [57], differentiation [54], neurodegeneration [58], and the stress response to oxygen deprivation [59].

#### Protein turnover and proteostasis

Protein homeostasis, or proteostasis, plays an important role in the pathogenesis of many diseases [60]. To counter adverse effects of oxidative stress, cells attempt to repair oxidized proteins by induction of chaperones, or by rapidly channeling damaged proteins into the ubiquitin-proteasome system (UPS) or autophagy for degradation [61,62]. The UPS, however, is the main protein degradation system in the heart, degrading up to 90% of intracellular proteins [63]. Accumulation of oxidized and misfolded proteins is a characteristic of cardiac disease and aging. Proteolysis-resistant proteins, referred to as soluble oligomeric forms of amyloidogenic proteins, exhibit cellular toxicity and may form large insoluble protein aggregates. Applying pathway analysis (Figs 4 and 5) indicated various proteins associated with ubiquitin C, small ubiquitin-like modifier (SUMO) 4 or amyloid precursor protein (APP). In addition, isoform-ß of heat shock protein (HSP) 105, which could be involved in preventing aggregation of denatured proteins [64,65], was less oxidized in Cat Tg mice (Hsph1, Q61699-2, 1.7-fold decrease in thiol occupancy of Cys167, Cat Tg *vs*. WT), potentially increasing or at least preserving its ability to repair altered proteins. Protein aggregates may also bind and deplete other functionally significant proteins, thus, preventing formation of these aggregates is critical to attenuating cardiac disease and aging.

### Functional effects of catalase overexpression on cardiac mitochondria in Cat Tg mice

**Fig 6:**
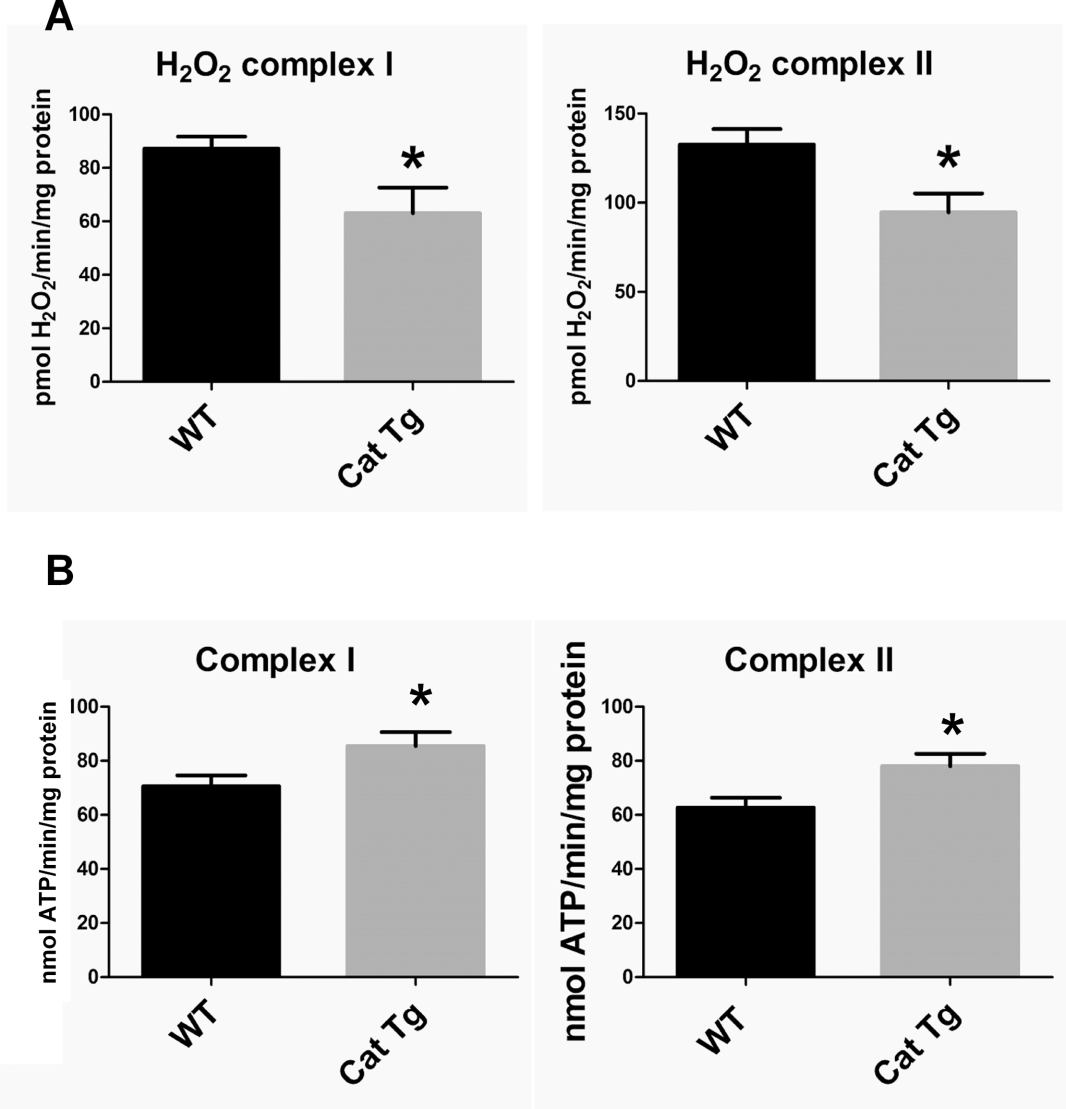
Cardiac mitochondrial H_2_O_2_ production and ATP synthesis rates in Cat Tg mice. (A) H_2_O_2_ production rates with a complex I substrate (5 mM pyruvate + 5 mM malate) and with a complex II substrate (5 mM succinate and inhibitor of reverse electron transport 2 μM rotenone); (B) Complex I (5 mM pyruvate + 5mM malate) and complex II (5 mM succinate + 2 μM rotenone) substrate-driven ATP synthesis rates. Data represents means ± SEM; N = 5–7; * P<0.05.

**Fig 7:**
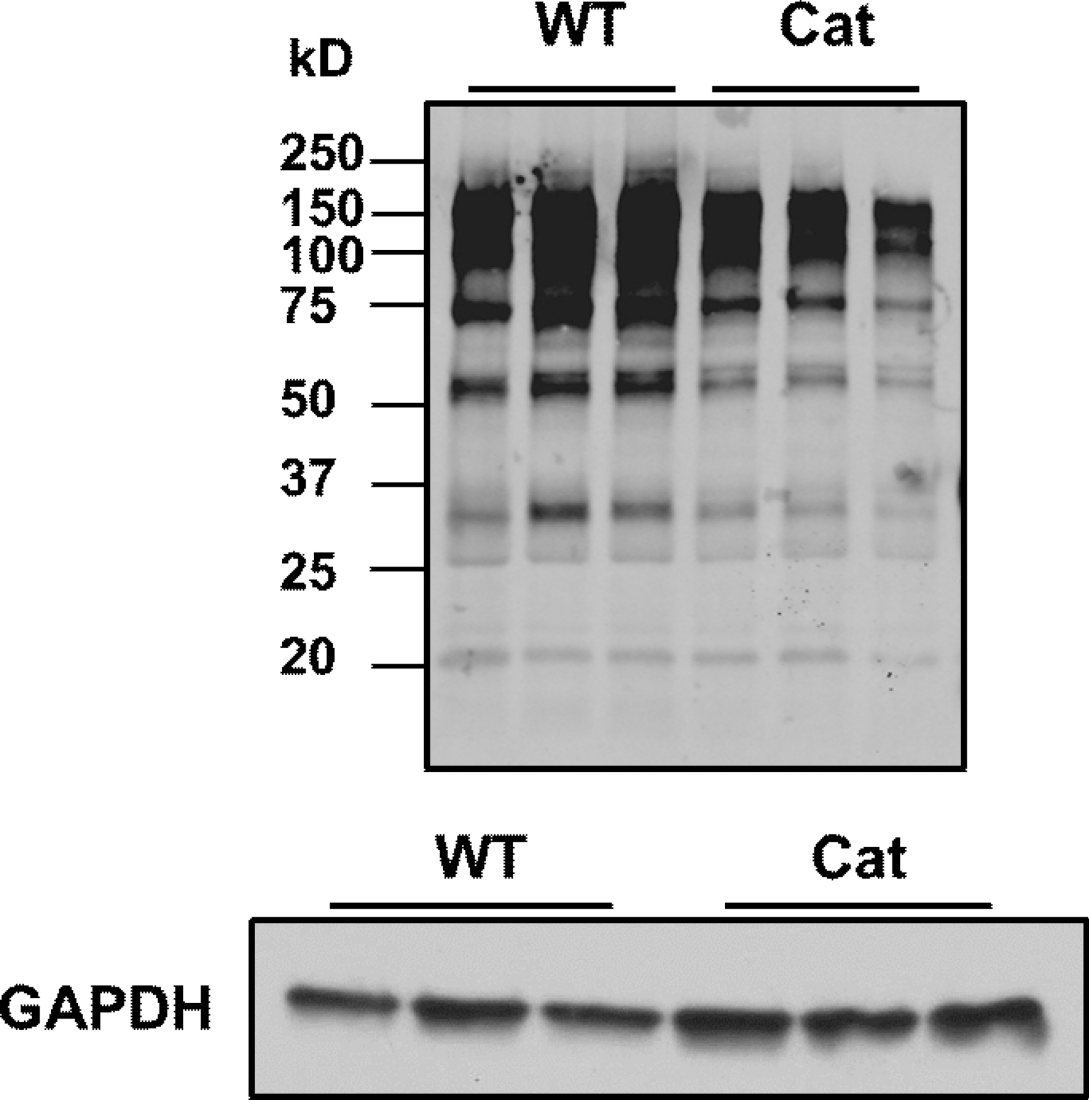
Protein-GSH adduct levels are decreased in catalase transgenic mice. Consistent with the decrease in H_2_O_2_ formation, protein GSH adducts were decreased in catalase transgenic mouse hearts, as measured by Western blotting analysis using an anti-GSH adduct antibody. GAPDH protein levels in hearts were analyzed by Western blot to ensure equal protein loading.

We previously reported that hearts of Cat Tg at 21 months of age were protected from an age-dependent decline in diastolic function and oxidative stress [4]. However, young and middle-aged wild type (WT) and Cat Tg mice revealed no apparent differences in cardiac diastolic function [4]. Because mitochondria can be a major cellular oxidant source [1], we measured mitochondrial oxygen consumption rates, ATP synthesis and H_2_O_2_ generation. Mitochondria isolated from adult Cat Tg mouse left ventricles produced significantly less H_2_O_2_ from complexes I and II (Fig 6A) and exhibited increased ATP synthesis rates when utilizing either complex I or II substrates (Fig 6B), sans significant differences in oxygen consumption rates (S4 Fig). This suggests that catalase overexpression could improve mitochondrial function by decreasing ROS generation while concomitantly increasing the efficiency of oxidative phosphorylation. Consistent with the decrease in mitochondrial-derived H_2_O_2_ in Cat Tg mice, protein GSH-adducts were globally diminished (Fig 7), suggesting that reversible cysteine oxidation detected by MS may be predominantly GSH-adducts. Furthermore, these data show that adult mice already exhibit significant changes in reversible protein cysteine thiol oxidation prior to an age-dependent decline in cardiac function. Catalase found in heart tissue has only about 2% of native specific activity exhibited by catalase in the liver. This may explain the heart’s vulnerability to basal levels of oxidants and the protective effects exhibited by cardiac catalase overexpression [66–68].

## Conclusion

Our proteomics screen to detect and quantify reversible cysteine thiol oxidation in the myocardium of adult Cat Tg mice identified various alterations that could be pertinent to cardiac disease and aging. Preserved cardiac diastolic function was apparent in the hearts of adult Cat Tg mice, yet we found a significant decrease in global reversible cysteine thiol oxidation, especially of mitochondrial proteins. Thus, we postulate that these changes in cysteine oxidation contribute to the protective effects eventually conferred by catalase overexpression in aged mice [10]. The oxidation status of major biological pathways involved in cardiac disease and aging was affected by catalase overexpression, including cellular stress response, proteostasis, antioxidant systems, apoptosis and myocardial contractile apparatus. We believe that this proteomics study identified novel target proteins that could be important for myocardial aging. Future studies are required, to confirm the functional significance of these protein targets.

## Supporting Information

S1 Fig Multiplexed quantitation of reversible cysteine oxidation in Cat Tg and WT mice. (A) TMT-switch labeling strategy. To determine the total available (free and reducible) cysteines, half of each left ventricle was completely reduced and labeled with iodoTMT; to label only reversibly oxidized cysteines, all free cysteines were blocked by IAM during lysis of the other half of the same left ventricles. Then those reversibly oxidized cysteines were reduced by TCEP prior to iodoTMT labeling. Tagged samples were pooled, precipitated to remove excess tag and detergent, and digested with trypsin. Immonopurified tagged peptides were analyzed using a Thermo Q Exactive MS/MS instrument. (B) MS data analysis strategy. From acquired MS/MS spectra, the thiol occupancy was calculated as the ratio between reporter ions used tag reversibly oxidized cysteines and those used to tag total available cysteines. TMT-tagged peptide/protein identification and quantitation from qualified mass spectra was performed in Proteome Discoverer 1.4. Proteins exhibiting changes in reversible cysteine oxidation were further studied for their roles in biological pathways and system biology.
(TIF)

S2 Fig Validation of TMT-tagged peptides. (A) Peptide frequency histogram of the coefficient of variation (CV) for changes in total available cysteine thiols (●), reversibly oxidized cysteine thiols (□) and their occupancy (∆). (B) Distribution of TMT-tagged peptides. A total of 2264 peptides with modifications were detected by LC-MS/MS analysis, of which 2125 peptides (94%) contained TMT-labeled cysteine thiols. The recovery of TMT-tagged peptide was greatly improved by removing unreacted TMT tag using protein precipitation prior to incubation with the TMT antibody resin. In summary, 1711 peptides (76%) of the peptides were single-, 348 peptides (15%) double-, and 66 peptides (3%) triple-tagged with TMT. While peptides containing a single cysteine enable quantification on a site-specific basis, peptides with multiple tagged cysteines only allow measuring average oxidation across all affected cysteines. (C) Overlap of TMT-tagged peptides exhibiting changes in total available (in white, left) and reversibly oxidized cysteines (in light grey, right) with CV≤35%. Quantitative proteome analysis requires stepwise selection of qualifying MS data (see methods for details). In general, reporter ions for total available cysteine thiols (m/z 127 or 129) were more abundant than those for reversibly oxidized cysteine thiols (m/z 126 or 128), resulting in better MS quantification and lower data variability as determined by the coefficient of variation (CV). By selecting a cutoff CV of ≤35% for all reporter ion ratios, adequate analytical precision was attained. A total of 658 peptides with quantification values for total available cysteine thiols and 285 peptides with quantification values for reversibly oxidized cysteine thiols qualified for further analysis. The union of both data sets contained 199 overlapping peptides, of which 109 peptides from 82 proteins exhibited ≥1.3-fold change in reversible cysteine oxidation. These 82 proteins were submitted for biological pathway analysis.
(TIF)

S3 Fig IPA predicted multiple protein networks associated with oxidative changes caused by catalase overexpression. The 11 ‘node’ proteins are highlighted in grey. Legend to network analysis: enzyme (diamond), transmembrane receptor (vertical oval), transcriptional regulator (horizontal oval), phosphatase (triangle), transporter (trapezoid), kinase (triangle), growth factor (square), and other (circle). Relationships: interaction (line), activation (arrow).
(TIF)

S4 Fig Cardiac mitochondrial maximal and uncoupled oxygen consumption were similar in both groups. **(**A) Maximal (State III) and uncoupled (oligomycin 2μM) (State IV) complex I substrate-driven oxygen consumption rate; (B) Maximal (State III) and uncoupled (oligomycin 2 μM) (State IV) complex II substrate-driven oxygen consumption rate. Data represents means ± SEM; N = 4–6.
(TIF)

S1 Table Complete list of proteins with a change in thiol oxidation in Cat Tg *vs*. WT. Accession number, gene ID, sites of modification and peptide sequences were retrieved from the Uniprot knowledgebase. Fold changes in Cat Tg *vs*. WT, were calculated from ratio of reporter ions for changes in total available cysteine as (m/z 129)/(m/z 127), reversibly oxidized cysteine thiols as (m/z 128)/(m/z 126) and the thiol occupancy as ((m/z 128)/ (m/z 126))/((m/z 129)/(m/z 127)). The thiol occupancy columns indicate percentage thiol occupancy, calculated as (m/z 126)/(m/z 127) for WT, and (m/z 128) /(m/z 129) for Cat Tg, together with The standard error mean (SEM) was calculated from N = 5 biological replicates.
(DOCX)

S2 Table Proteins with a change in thiol occupancy by >2-fold in Cat Tg *vs*. WT mice. Accession number, gene ID, sites of modification and peptide sequences were retrieved from the Uniprot knowledgebase. Fold changes in Cat Tg *vs*. WT, were calculated from ratio of reporter ions for changes in total available cysteine as (m/z 129)/(m/z 127), reversibly oxidized cysteine thiols as (m/z 128)/(m/z 126) and the thiol occupancy as ((m/z 128)/ (m/z 126))/((m/z 129)/(m/z 127)). The thiol occupancy columns indicate percentage thiol occupancy, calculated as (m/z 126)/(m/z 127) for WT, and (m/z 128) /(m/z 129) for Cat Tg, together with The standard error mean (SEM) was calculated from N = 5 biological replicates.
(DOCX)

S3 Table Proteins involved in five canonical pathways predicted in IPA, with a change in thiol occupancy by >1.3-fold in Cat Tg *vs*. WT. Accession number, gene ID, sites of modification and peptide sequences were retrieved from the Uniprot knowledgebase. Fold changes in Cat Tg *vs*. WT, were calculated from ratio of reporter ions for changes in total available cysteine as (m/z 129)/(m/z 127), reversibly oxidized cysteine thiols as (m/z 128)/(m/z 126) and the thiol occupancy as ((m/z 128)/ (m/z 126))/((m/z 129)/(m/z 127)). The thiol occupancy columns indicate percentage thiol occupancy, calculated as (m/z 126)/(m/z 127) for WT, and (m/z 128) /(m/z 129) for Cat Tg, together with The standard error mean (SEM) was calculated from N = 5 biological replicates.
(DOCX)

## Abbreviations

7-kDa MWCO: 7-kDa molecular weight cutoff
ACN: acetonitrile
Cat Tg: catalase overexpressing transgenic mouse
CV: coefficient of variation
Cys: cysteine
DPTA: diethylene triamine pentaacetic acid
DTT: dithiothreitol
ECM: extracellular matrix
FDR: false discovery rate
FA: formic acid
fwhm: full width at half maximum
GSH: glutathione
HCD: higher-energy collisional dissociation
H_2_O_2_: hydrogen peroxide
IP: immunoprecipitation
IPA: Ingenuity Pathway Analysis
IAM: iodoacetamide
iodoTMT: iodoacetyl reactive tandem mass tags
LV: left ventricle of the heart
LC-MS/MS: liquid chromatography-tandem MS
MS: mass spectrometry
NCE: normalized collision energy
PD: Proteome Discoverer
SDS-PAGE: sodium dodecyl sulfate polyacrylamide gel electrophoresis
TFA: trifluoroacetic acid
TBS: Tris-buffered saline
TCEP: Tris(2-carboxyethyl)phosphine
UPS: ubiquitin-proteasome system
WT: wild type
