# Supplementary figures and images for: Overexpression of Catalase Diminishes Oxidative Cysteine Modifications of Cardiac Proteins
Source: PLoS One. 2015 Dec 7;10(12):e0144025. doi: 10.1371/journal.pone.0144025 (PMC4671598; doi:10.1371/journal.pone.0144025)

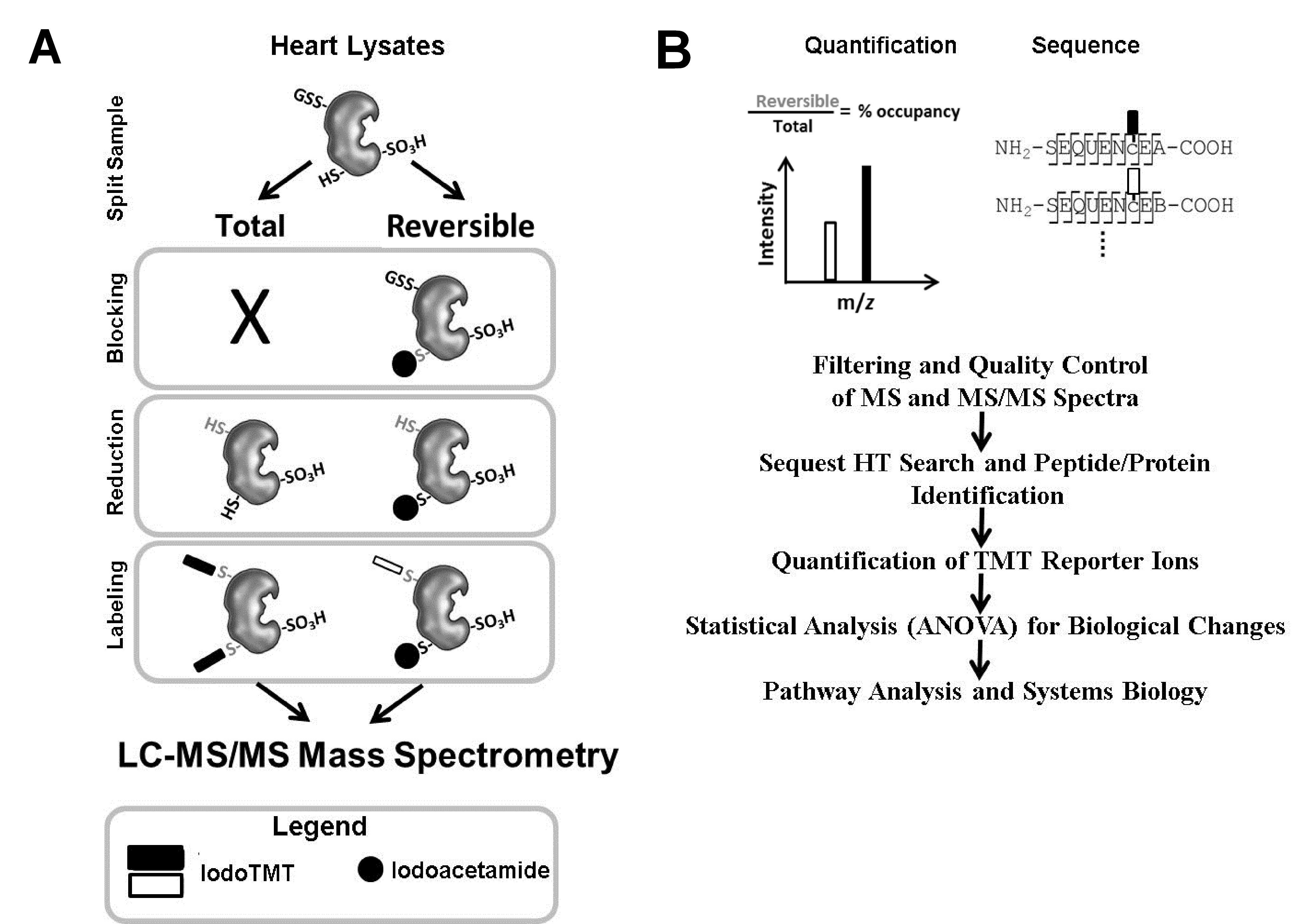

Supplement: S1 Fig — (A) TMT-switch labeling strategy. To determine the total available (free and reducible) cysteines, half of each left ventricle was completely reduced and labeled with iodoTMT; to label only reversibly oxidized cysteines, all free cysteines were blocked by IAM during lysis of the other half of the same left ventricles. Then those reversibly oxidized cysteines were reduced by TCEP prior to iodoTMT labeling. Tagged samples were pooled, precipitated to remove excess tag and detergent, and digested with trypsin. Immonopurified tagged peptides were analyzed using a Thermo Q Exactive MS/MS instrument. (B) MS data analysis strategy. From acquired MS/MS spectra, the thiol occupancy was calculated as the ratio between reporter ions used tag reversibly oxidized cysteines and those used to tag total available cysteines. TMT-tagged peptide/protein identification and quantitation from qualified mass spectra was performed in Proteome Discoverer 1.4. Proteins exhibiting changes in reversible cysteine oxidation were further studied for their roles in biological pathways and system biology. (TIF) [file pone.0144025.s001.tif]

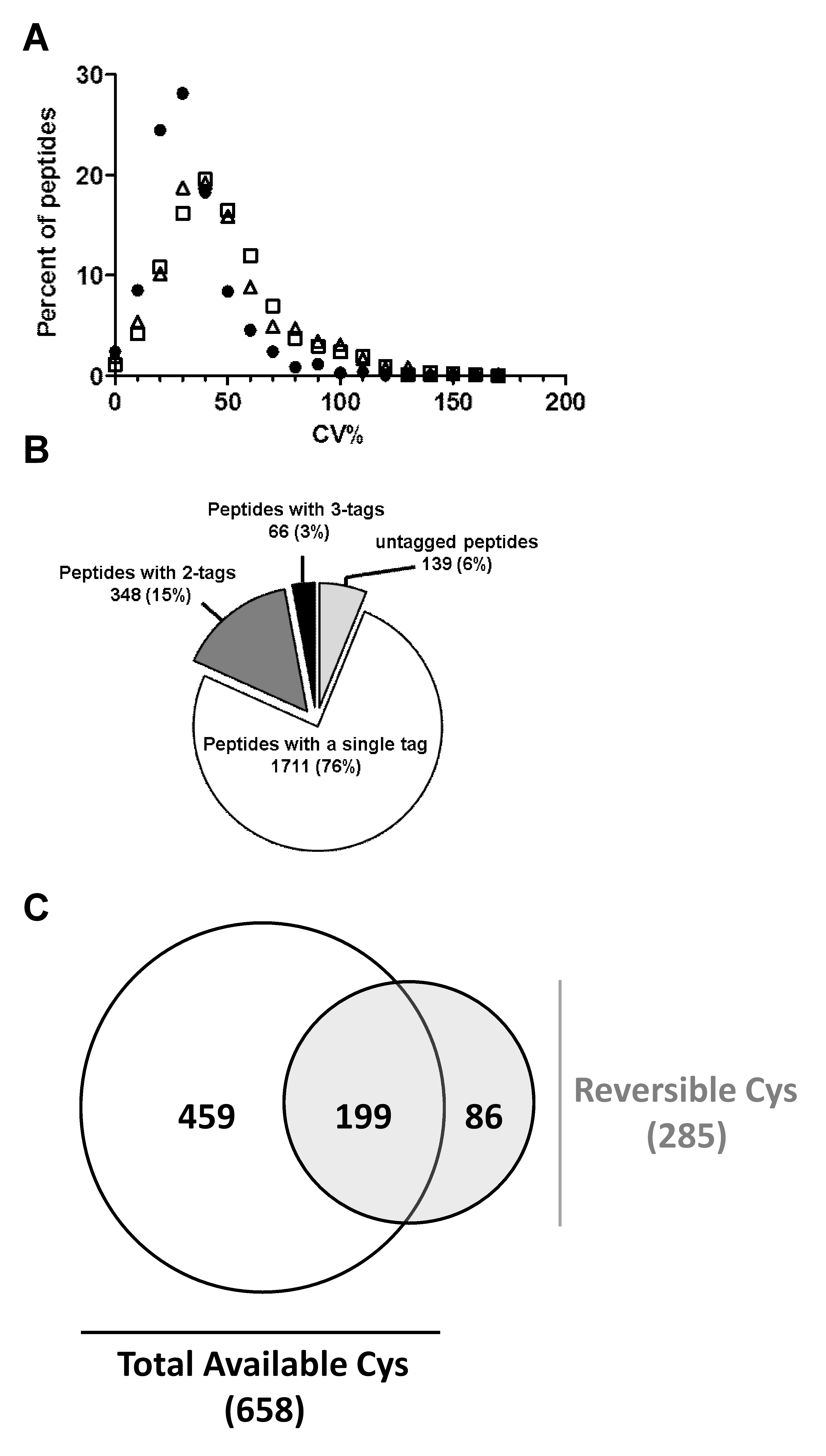

Supplement: S2 Fig — (A) Peptide frequency histogram of the coefficient of variation (CV) for changes in total available cysteine thiols (●), reversibly oxidized cysteine thiols (□) and their occupancy (∆). (B) Distribution of TMT-tagged peptides. A total of 2264 peptides with modifications were detected by LC-MS/MS analysis, of which 2125 peptides (94%) contained TMT-labeled cysteine thiols. The recovery of TMT-tagged peptide was greatly improved by removing unreacted TMT tag using protein precipitation prior to incubation with the TMT antibody resin. In summary, 1711 peptides (76%) of the peptides were single-, 348 peptides (15%) double-, and 66 peptides (3%) triple-tagged with TMT. While peptides containing a single cysteine enable quantification on a site-specific basis, peptides with multiple tagged cysteines only allow measuring average oxidation across all affected cysteines. (C) Overlap of TMT-tagged peptides exhibiting changes in total available (in white, left) and reversibly oxidized cysteines (in light grey, right) with CV≤35%. Quantitative proteome analysis requires stepwise selection of qualifying MS data (see methods for details). In general, reporter ions for total available cysteine thiols (m/z 127 or 129) were more abundant than those for reversibly oxidized cysteine thiols (m/z 126 or 128), resulting in better MS quantification and lower data variability as determined by the coefficient of variation (CV). By selecting a cutoff CV of ≤35% for all reporter ion ratios, adequate analytical precision was attained. A total of 658 peptides with quantification values for total available cysteine thiols and 285 peptides with quantification values for reversibly oxidized cysteine thiols qualified for further analysis. The union of both data sets contained 199 overlapping peptides, of which 109 peptides from 82 proteins exhibited ≥1.3-fold change in reversible cysteine oxidation. These 82 proteins were submitted for biological pathway analysis. (TIF) [file pone.0144025.s002.tif]

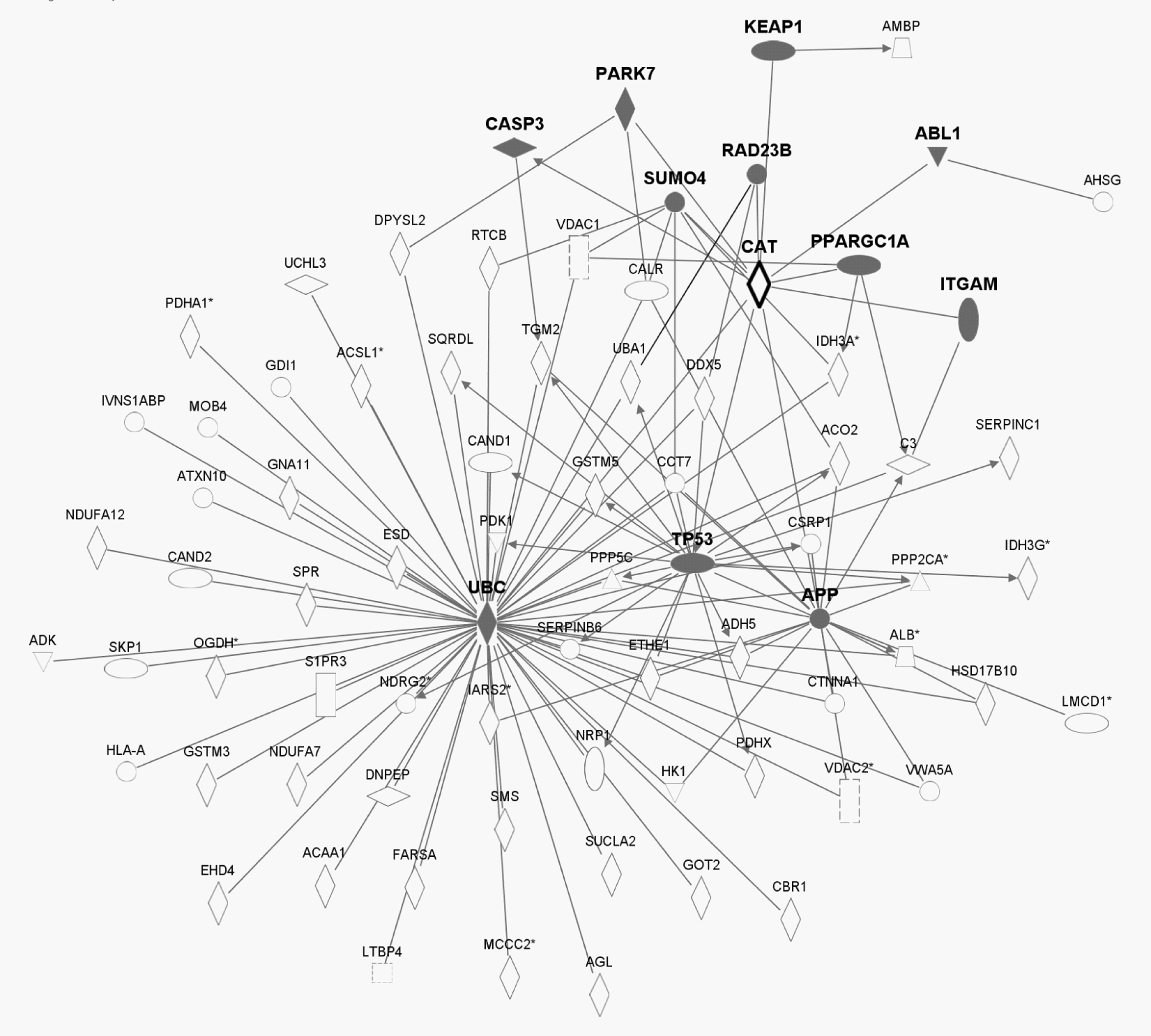

Supplement: S3 Fig — The 11 ‘node’ proteins are highlighted in grey. Legend to network analysis: enzyme (diamond), transmembrane receptor (vertical oval), transcriptional regulator (horizontal oval), phosphatase (triangle), transporter (trapezoid), kinase (triangle), growth factor (square), and other (circle). Relationships: interaction (line), activation (arrow). (TIF) [file pone.0144025.s003.tif]

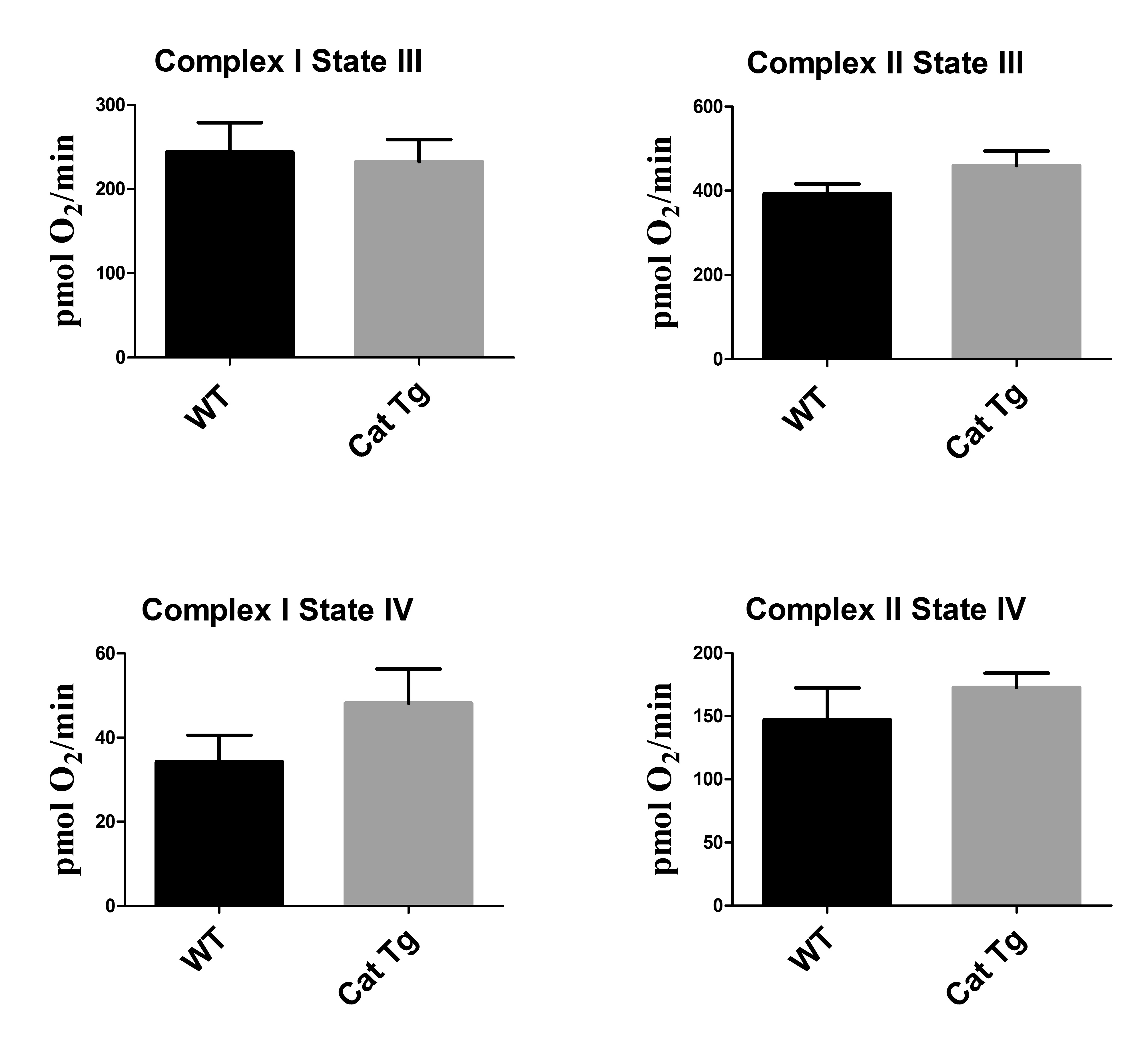

Supplement: S4 Fig — (A) Maximal (State III) and uncoupled (oligomycin 2μM) (State IV) complex I substrate-driven oxygen consumption rate; (B) Maximal (State III) and uncoupled (oligomycin 2 μM) (State IV) complex II substrate-driven oxygen consumption rate. Data represents means ± SEM; N = 4–6. (TIF) [file pone.0144025.s004.tif]
