# Supplementary material for: Overexpression of Catalase Diminishes Oxidative Cysteine Modifications of Cardiac Proteins
Source: PLoS One. 2015 Dec 7;10(12):e0144025. doi: 10.1371/journal.pone.0144025 (PMC4671598; doi:10.1371/journal.pone.0144025)
Supplement: S1 Table — Accession number, gene ID, sites of modification and peptide sequences were retrieved from the Uniprot knowledgebase. Fold changes in Cat Tg vs. WT, were calculated from ratio of reporter ions for changes in total available cysteine as (m/z 129)/(m/z 127), reversibly oxidized cysteine thiols as (m/z 128)/(m/z 126) and the thiol occupancy as ((m/z 128)/ (m/z 126))/((m/z 129)/(m/z 127)). The thiol occupancy columns indicate percentage thiol occupancy, calculated as (m/z 126)/(m/z 127) for WT, and (m/z 128) /(m/z 129) for Cat Tg, together with The standard error mean (SEM) was calculated from N = 5 biological replicates. (DOCX) [file pone.0144025.s005.docx]

| **Accession No.** | **GN** | **Protein description** | **Cys sites** | **Sequence** | **Fold changes (Cat Tg vs WT)** | | | **Occupancy± SEM (%)** | |
| --- | --- | --- | --- | --- | --- | --- | --- | --- | --- |
|  |  |  |  |  |  |  |  |  |  |
|  |  |  |  |  | **Total available Cys** | **Reversibly oxidized Cys** | **Cys thiol occupancy** | **WT** | **Cat Tg** |
| Q3UPU8 | Acaa1a | 3-ketoacyl-CoA thiolase A, peroxisomal | C177 | DcLTPMGMTSENVAER | 1.5 | -1.5 | -2.1 | 36.8±3.9 | 17.9±1.9 |
| Q9D7B6 | Acad8 | Isobutyryl-CoA dehydrogenase, mitochondrial | C157 | FASYcLTEPGSGSDAASLLTSAK | 1.1 | -2.6 | -3.2 | 10.1±2.1 | 2.5±0.6 |
| P51174 | Acadl | Long-chain specific acyl-CoA dehydrogenase, mitochondrial | C166 | cIGAIAmTEPGAGSDLQGVR | 1.1 | -1.2 | -1.3 | 70.3±6.8 | 53.6±4.9 |
|  |  |  | C166 | cIGAIAMTEPGAGSDLQGVR | 1.1 | -1.1 | -1.2 | 85.3±8 | 70±6.2 |
|  |  |  | C351 | AFVDScLQLHETK | -1 | -1.1 | -1 | 65.4±11.4 | 55.7±5.4 |
| D3Z2A5 | Acadm | Medium-chain-specific acyl-CoA dehydrogenase, mitochondrial (Fragment) | C124;C127 | mTEQPMMcAYcVTEPSAGSDVAAIK | 1.1 | -1.2 | -1.3 | 74.2±5.1 | 55.9±4.8 |
|  |  |  | C124;C127 | MTEQPMMcAYcVTEPSAGSDVAAIK | 1.1 | -1.2 | -1.3 | 80.6±5.9 | 60.5±5.1 |
|  |  |  | C124;C127 | MTEQPmmcAYcVTEPSAGSDVAAIK | 1 | -1.2 | -1.2 | 69.4±6 | 53.9±4.7 |
| Q7TMY2 | Acadsb | Acyl-Coenzyme A dehydrogenase, short/branched chain | C385 | cIEWMGGVGYTK | 1 | -1.2 | -1.2 | 31.3±2.5 | 22.1±3.4 |
| Q99KI0 | Aco2 | Aconitate hydratase, mitochondrial | C385 | VGLIGScTNSSYEDmGR | 1.1 | -1.4 | -1.4 | 43.4±6.1 | 28.9±5 |
| D3Z041 | Acsl1 | Long-chain-fatty-acid--CoA ligase 1 | C55 | ALKPPcDLSMQSVEIAGTTDGIR | 1.1 | -1.2 | -1.5 | 11.5±2.4 | 6.4±1.2 |
|  |  |  | C626 | GLQGSFEELcR | 1.2 | -1.3 | -1.5 | 24.3±2.8 | 16.5±3.2 |
|  |  |  | C275 | VKPKPPEPEDLAIIcFTSGTTGNPK | 1.2 | -1.2 | -1.5 | 36.9±6.3 | 24.9±4.8 |
|  |  |  | C298 | GAmITHQNIINDcSGFIK | 1.2 | -1.3 | -1.5 | 39.9±6.5 | 26±3.8 |
|  |  |  | C109 | GIQVSNNGPcLGSR | 1.2 | -1.2 | -1.4 | 46.7±8 | 34.1±5.7 |
|  |  |  | C298 | GAMITHQNIINDcSGFIK | 1.2 | -1.2 | -1.3 | 51.4±9.1 | 36.3±5.7 |
| P28474 | Adh5 | Alcohol dehydrogenase class-3 | C240 | EFGASEcISPQDFSK | 1.1 | -1.4 | -1.5 | 41.7±2.7 | 29.7±3.9 |
| Q8BMC5 | Adk | Adenosine kinase | C159 | SLVANLAAANcYK | 1.1 | -1.6 | -1.8 | 33.6±2.1 | 19.6±2.7 |
| E9Q4C0 | Adprhl1 | [Protein ADP-ribosylarginine] hydrolase-like protein 1 (Fragment) | C151 | VLPLAEEYcR | 1 | -1.4 | -1.3 | 20.7±1 | 16.6±2.7 |
| F8VPN4 | Agl | Protein Agl | C81 | SLDWENPTEREDDSDKYcK | 1 | -1.8 | -1.8 | 80.8±3.7 | 45.4±6.2 |
| P29699 | Ahsg | Alpha-2-HS-glycoprotein | C114 | QLTEHAVEGDcDFHILK | 1.2 | -1.5 | -1.8 | 56.3±3.5 | 31.4±4.1 |
| P07724 | Alb | Serum albumin | C591 | DTcFSTEGPNLVTR | 1.1 | -1.8 | -1.8 | 92.5±18.4 | 43.3±5.8 |
|  |  |  | C269;C270;C277 | VNKEccHGDLLEcADDRAELAK | 1.1 | -1.4 | -1.5 | 30.7±1.2 | 20.4±1.9 |
|  |  |  | C269;C270;C277 | EccHGDLLEcADDRAELAK | 1.1 | -1.3 | -1.5 | 35.1±4.4 | 23.5±1.8 |
|  |  |  | C269;C270;C277 | VNKEccHGDLLEcADDR | 1.1 | -1.2 | -1.4 | 31.6±3.5 | 22.4±2.2 |
| Q07456 | Ambp | Protein AMBP | C336 | EYcGVPGDGYEELIR | 1.1 | -1.4 | -1.6 | 62.7±3.6 | 42.6±6.1 |
| Q3TE53 | Anks1 | Ankyrin repeat and SAM domain-containing protein 1A | C209;C226 | cQDLISQTSSPLSQNDScTGR | 1.2 | -1.3 | -1.4 | 17.4±3.7 | 13.2±4.3 |
| B7STB7 | Anxa1 | Annexin | C343 | ILVALcGGN | 1.3 | -1.2 | -1.5 | 19.2±3.5 | 12.8±2.8 |
| Q8K4Z3 | Apoa1bp | NAD(P)H-hydrate epimerase | C109;C121 | SPPTVLVIcGPGNNGGDGLVcAR | 1 | -1.2 | -1.1 | 47.9±5.6 | 42.6±6.8 |
| Q4FZG5 | Arpc2 | Arpc2 protein (Fragment) | C96 | NcFASVFEK | 1.6 | -1.1 | -1.7 | 47.3±4.2 | 27±1.5 |
| Q8BWX1 | Atxn10 | Ataxin-10 | C283 | HAELIANSFMDQcR | 1.1 | -1.5 | -1.4 | 30.9±3.3 | 22.7±8.2 |
| E9Q705 | Bola3 | BolA-like protein 3 | C47 | ATAIQVTDISGGcGAMYEIK | 1 | -2.9 | -2.9 | 10.6±1 | 3.8±0.6 |
| P01027 | C3 | Complement C3 | C1513;C1518 | cAEENcFMQQSQEK | 1.2 | -1.5 | -1.8 | 52.1±16.7 | 29.6±10.8 |
|  |  |  | C559 | DScIGTLVVK | 1 | -1.2 | -1.2 | 57.5±1.3 | 45.6±5.7 |
|  |  |  | C1158 | DIcEGQVNSLPGSINK | 1.1 | -1.1 | -1.2 | 48.2±1.9 | 42±5.1 |
| A2A998 | C8a | Complement component 8, alpha polypeptide | C71;C77;C86 | HLVcNGDNDcLDGSDESDcEDVR | 1.7 | -1.1 | -1.8 | 117.6±0.8 | 64.3±10.7 |
|  |  |  | C133 | YYGGQcETVYNGDWR | 1.4 | 1 | -1.4 | 66.7±17.5 | 44.8±9.6 |
| Q3UWP8 | Calr | Calreticulin | C105 | HEQNIDcGGGYVK | -1 | -1.4 | -1.3 | 84.5±14.7 | 69.2±15.6 |
| Q3UMI9 | Cand1 | Cullin-associated NEDD8-dissociated protein 1 | C237 | TYIQcIAAISR | 1.1 | -2.2 | -2.3 | 19.7±1.2 | 8.7±1.4 |
| Q3UM57 | Cand2 | Cullin-associated NEDD8-dissociated protein 2 | C131 | TVLSELPPAATGSGLAINVcR | 1.1 | -2 | -1.7 | 12.8±3.5 | 7.5±2.1 |
| Q3UF58 | Cat | Catalase | C376 | LGPNYLQIPVNcPYR | 14 | -1.7 | -24.5 | 35.5±3.8 | 1.9±0.3 |
| P48758 | Cbr1 | Carbonyl reductase [NADPH] 1 | C226;C227 | ILLNAccPGWVR | 1.4 | -2.1 | -3 | 17.9±5.3 | 6.2±2.7 |
| Q3UDB1 | Cct7 | T-complex protein 1 subunit eta | C450 | QLcDNAGFDATNILNK | 1.2 | -1.4 | -1.8 | 37.1±3.9 | 21±4.3 |
| Q9CS06 | Cct8 | T-complex protein 1 subunit theta | C417 | QITSYGETcPGLEQYAIK | 1.3 | -1.2 | -1.5 | 50.8±5.4 | 32.7±3 |
| B8JJM3 | Cfb | Complement factor B (Fragment) | C176;C189 | FLcTGGVDPYADPNTcK | 1.2 | -1.4 | -1.6 | 79±2.5 | 45.6±4.7 |
| Q6P8J7 | Ckmt2 | Creatine kinase S-type, mitochondrial | C63 (IAM);C67 | HNNcMAEcLTPTIYAK | 1.2 | -1.1 | -1.4 | 370.3±61.7 | 335.2±46.5 |
|  |  |  | C180 | GLSLPPAcSR | 1 | -1.4 | -1.3 | 53.5±5.8 | 42±10.1 |
|  |  | Creatine kinase S-type | C63;C67(diOxi) | HNNcMAEcLTPTIYAK | -1.1 | -1.4 | -1.3 | 54.3±4.3 | 42.1±0 |
|  |  | (continued) | C90 | mTPSGYTLDQcIQTGVDNPGHPFIK | 1 | -1.3 | -1.2 | 64.4±8.1 | 49.9±8 |
|  |  |  | C63;C67 | HNNcmAEcLTPTIYAK | 1 | -1.2 | -1.2 | 19.1±4.7 | 13.2±2.5 |
|  |  |  | C90 | MTPSGYTLDQcIQTGVDNPGHPFIK | -1 | -1.3 | -1.2 | 69.3±7.7 | 56.6±8.6 |
| Q8R4N0 | Clybl | Citrate lyase subunit beta-like protein, mitochondrial | C67;C72 | VDcAVLDcEDGVAENK | 1.1 | -2 | -2.1 | 46.3±7.6 | 19.8±2.9 |
|  |  |  | C67;C72 | VDcAVLDcEDGVAENKK | -1 | -1.3 | -1.1 | 21.8±0.6 | 19.9±2.2 |
| Q3U5V2 | Cnbp | Cellular nucleic acid-binding protein | C60(IAM);C68;C71 | DcDLQEDEAcYNcGR | 1.4 | -1.1 | -1.6 | 272.9±7.8 | 172.9±30.6 |
| P56391 | Cox6b1 | Cytochrome c oxidase subunit 6B1 | C30 | NcWQNYLDFHR | 1.2 | -1.1 | -1.3 | 48.4±3.8 | 36.9±4.2 |
|  |  |  | C54 | GGDVSVcEWYR | 1.2 | -1.1 | -1.2 | 41.5±4.5 | 32.7±3.5 |
|  |  |  | C65 | SLcPVSWVSAWDDR | 1.1 | 1.1 | -1 | 52±5.5 | 49.2±6.4 |
| Q3UIM5 | Cpt1b | Carnitine O-palmitoyltransferase 1, muscle isoform | C526 | LPWDIPEQcR | 1.1 | -1.3 | -1.3 | 31.8±6.4 | 22.6±3.4 |
| Q80X68 | Csl | Citrate synthase | C101 | GYSIPEcQK | -1.1 | -1.1 | 1 | 65.1±9.9 | 66.5±9.3 |
| P97315 | Csrp1 | Cysteine and glycine-rich protein 1 | C58 | NLDSTTVAVHGEEIYcK | 1 | -4.1 | -4.2 | 24±2.8 | 5.7±1 |
|  |  |  | C25 | TVYFAEEVQcEGNSFHK | 1 | -1.2 | -1.1 | 41.2±10.4 | 33.5±5 |
| P50462 | Csrp3 | Cysteine and glycine-rich protein 3 | C79 | GIGFGQGAGcLSTDTGEHLGLQFQQSPKPAR | -1 | -3.1 | -3 | 6.6±0.8 | 4.4±1.4 |
|  |  |  | C168 | SLESTNVTDKDGELYcK | 1 | -1.4 | -1.4 | 4.7±0.5 | 9±0.5 |
| P26231 | Ctnna1 | Catenin alpha-1 | C324 | LESIISGAALMADSScTR | 1.2 | -1.8 | -1.8 | 3.9±0.6 | 2.9±0.8 |
| P10605 | Ctsb | Cathepsin B | C319 | GENHcGIESEIVAGIPR | 1.2 | -1.3 | -1.5 | 53.3±6.4 | 36.2±4.5 |
|  |  |  | C93 | EQWSNcPTIGQIR | 1.2 | -1.2 | -1.4 | 88.3±5.2 | 63.2±5.9 |
| Q3U7I9 | Ctsd | Cathepsin D | C321 | AIGAVPLIQGEYMIPcEK | 1.2 | -1.2 | -1.3 | 34.3±2.8 | 23.5±2.1 |
| Q3TJ01 | D10Wsu52e | tRNA-splicing ligase RtcB homolog | C485 | NVTDVVNTcHDAGISK | 1.1 | -1.4 | -1.6 | 24.5±5.8 | 17.1±6 |
| Q61656 | Ddx5 | Probable ATP-dependent RNA helicase DDX5 | C191 | ELAQQVQQVAAEYcR | 1.2 | -1.5 | -1.7 | 25.6±1.9 | 15.4±5.6 |
| Q3U0B3 | Dhrs11 | Dehydrogenase/reductase SDR family member 11 | C55 | TVGNIEELAAEcK | 1 | -1.6 | -1.6 | 26.6±2.8 | 16.4±2.5 |
| Q8BPW9 | Dnpep | Aspartyl aminopeptidase | C411 | NDSPcGTTIGPILASR | -1 | -1.6 | -1.5 | 27.6±0.9 | 17.8±1.2 |
| O08553 | Dpysl2 | Dihydropyrimidinase-related protein 2 | C439 | THNSALEYNIFEGMEcR | 1.2 | -1.4 | -1.6 | 61.4±12.1 | 37.5±4.5 |
| Q3TQ74 | Ehd4 | EH domain-containing protein 4 | C141 | FMcSQLPNQVLK | 1.3 | -1.7 | -2.2 | 17.8±1.9 | 8.4±1.4 |
| Q8BGY2 | Eif5a2 | Eukaryotic translation initiation factor 5A-2 | C73 | YEDIcPSTHNMDVPNIK | 1.1 | 1.1 | 1 | 21.4±6.3 | 21.5±5.2 |
| H3BJP2 | Esd | S-formylglutathione hydrolase (Fragment) | C206 | AYDATcLVK | 1 | -1.6 | -1.7 | 19.6±2.6 | 13.9±1.2 |
| Q9DCM0 | Ethe1 | Protein ETHE1, mitochondrial | C34 | ScTYTYLLGDR | 1 | -1.3 | -1.3 | 57.9±3.1 | 44.4±4.2 |
| H7BX99 | F2 | Prothrombin | C214 | DNLSPPLGQcLTER | 1.2 | -1.6 | -1.8 | 56.1±4.1 | 32.8±5.7 |
|  |  |  | C546 | ITDNMFcAGFK | 1 | -1.5 | -1.6 | 45.5±5.1 | 29.3±5.1 |
| E9PWY9 | Farsa | Phenylalanine--tRNA ligase alpha subunit | C492 | VNLQMVYDSPVcR | 1.3 | -1.4 | -1.7 | 20.8±7.9 | 10.4±2.5 |
| Q9WVH9 | Fbln5 | Fibulin-5 | C59;C68 | GDMMcVNQNGGYLcIPR | 1.2 | -1.4 | -1.6 | 80±14.4 | 45.8±1.6 |
| A2AQ53 | Fbn1 | Fibrillin 1 | C536 | DIDEcLQnGR | 1.3 | -1.3 | -1.6 | 101.6±10.9 | 63.3±12.2 |
|  |  |  | C639;C641 | cEcFPGLAVGLDGR | 1.2 | -1.2 | -1.4 | 38.8±5.1 | 27.7±6.5 |
| D6RCG1 | Gart | Phosphoribosylamine--glycine ligase | C237 | LLDGDEGPNTGGMGAYcPAPQVSK | 1.1 | -1.1 | -1.2 | 18.6±5.8 | 14.9±4.4 |
| O55126 | Gbas | Protein NipSnap homolog 2 | C88 | IcQEVLPK | -1 | -1.3 | -1.2 | 27.8±5.3 | 23.5±3.8 |
| P50396 | Gdi1 | Rab GDP dissociation inhibitor alpha | C317 | NTNDANScQIIIPQNQVNR | 1.3 | -1.3 | -1.6 | 121.1±8.4 | 73.8±3.6 |
| D3YWB5 | Gimap4 | GTPase IMAP family member 4 | C103 | MYESAEcVIQK | 1.2 | -1.3 | -1.5 | 28±1.5 | 18.5±3 |
| D3Z1B2 | Gm4953 | MCG50540 | ;C101 | ncAEFVSGSQLR | 1.5 | -2.2 | -3.2 | 32.1±8.1 | 9±1.7 |
| P21278 | Gna11 | Guanine nucleotide-binding protein subunit alpha-11 | C144 | TLWSDPGVQEcYDR | 1.1 | -1.3 | -1.4 | 46±4.8 | 33.1±4 |
| P08752 | Gnai2 | Guanine nucleotide-binding protein G(i) subunit alpha-2 | C255 | LFDSIcNNK | 1.2 | -1.1 | -1.3 | 37.6±10.5 | 27.8±7 |
| P68040 | Gnb2l1 | Guanine nucleotide-binding protein subunit beta-2-like 1 | C153 | YTVQDESHSEWVScVR | 1.1 | -1.2 | -1.4 | 51.8±6.2 | 37±4.2 |
|  |  |  | C207 | TNHIGHTGYLNTVTVSPDGSLcASGGK | 1.1 | -1.2 | -1.3 | 69.9±6.4 | 49.4±4.8 |
| F7ALS6 | Got1 | Aspartate aminotransferase, cytoplasmic (Fragment) | C122 | DIRPYcYWDAEK | -1 | -1.1 | -1.1 | 32.9±3.8 | 31.4±5.8 |
| P05202 | Got2 | Aspartate aminotransferase, mitochondrial | C187 | TcGFDFSGALEDISK | -1 | -1.4 | -1.3 | 48.2±6.1 | 35.1±5 |
| Q3ULJ0-2 | Gpd1l | Isoform 2 of Glycerol-3-phosphate dehydrogenase 1-like protein | C216 | NIVAVGAGFcDGLR | 1.3 | -1.3 | -1.5 | 25.6±5.7 | 17.3±4.4 |
| O70325-2 | Gpx4 | Isoform Cytoplasmic of Phospholipid hydroperoxide glutathione peroxidase, mitochondrial | C75 | ILAFPcNQFGR | 1.1 | -1.2 | -1.4 | 13.8±1.2 | 9.8±0.2 |
| P10649 | Gstm1 | Glutathione S-transferase Mu 1 | C115 | mQLIMLcYNPDFEK | 1.1 | -1.4 | -1.5 | 28.2±1.4 | 19.1±2 |
| E9PVM7 | Gstm5 | Glutathione S-transferase Mu 5 (Fragment) | C177 | cLDEFPNLK | 1.1 | -1.3 | -1.4 | 44.9±3.2 | 33.1±4.5 |
| Q61642 | H2-K1 | H-2K-sm1 | C357 | GGDYALAPGSQTSDLSLPDcK | 1.4 | -1.8 | -2.3 | 42.3±4.3 | 17.7±5.4 |
| G3UVV4 | Hk1 | Hexokinase 1, isoform CRA_f | C605 | MPLGFTFSFPcK | 1.2 | -1.3 | -1.5 | 15.5±2.5 | 11.7±1.6 |
| O08756 | Hsd17b10 | 3-hydroxyacyl-CoA dehydrogenase type-2 | C58 | LGEScIFAPANVTSEK | 1.1 | -1.6 | -1.6 | 16±3.6 | 10.7±3.9 |
| Q504P4 | Hspa8 | Heat shock cognate 71 kDa protein | C17 | GPAVGIDLGTTYScVGVFQHGK | 1.1 | -1.2 | -1.3 | 51.4±6.5 | 39±4.9 |
| B1B0C7 | Hspg2 | Endorepellin | C3409 | NIGASVEFHcAVPNER | 1.1 | -1 | -1.2 | 82.1±10.7 | 68.2±6.7 |
| Q61699-2 | Hsph1 | Isoform HSP105-beta of Heat shock protein 105 kDa | C167 | SVLDAAQIVGLNcLR | 1.7 | -1.1 | -1.8 | 52±13.1 | 32.2±14.7 |
| Q8BIJ6 | Iars2 | Isoleucine--tRNA ligase, mitochondrial | C465 | EENIVHSYPcDWR | 1.1 | -2.1 | -2.4 | 15.4±1.7 | 6.5±0.9 |
|  |  |  | C155 | VHFVPGWDcHGLPIETK | 1.1 | -1.4 | -1.5 | 53±1.8 | 35.5±5.7 |
| Q9D6R2-2 | Idh3a | Isoform 2 of Isocitrate dehydrogenase [NAD] subunit alpha, mitochondrial | C273;C281 | cSDFTEEIcR | 1.1 | -3.6 | -4 | 6±0.7 | 1.7±0.2 |
|  |  |  | C49 | TFDLYANVRPcVSIEGYK | 1.1 | -1.9 | -2.1 | 14.3±0.9 | 7±1.1 |
| Q684I8 | Idh3g | Isocitrate dehydrogenase 3 (NAD+), gamma (Fragment) | C146 | TSLDLYANVIHcK | 1.2 | -1.5 | -1.8 | 20.8±2.6 | 11.8±1.4 |
|  |  |  | C233;C234 | LGDGLFLQccR | 1.1 | -1.6 | -1.7 | 20±2.6 | 11.7±1.8 |
|  |  |  | C79 | HAcVPVDFEEVHVSSnADEEDIR | 1.3 | -1.3 | -1.6 | 23.6±2.8 | 14.4±0.4 |
| Q9DCB8 | Isca2 | Iron-sulfur cluster assembly 2 homolog, mitochondrial | C79 | LQVEGGGcSGFQYK | 1 | -1.3 | -1.4 | 24.4±2 | 16.1±0.8 |
| Q9JHI5 | Ivd | Isovaleryl-CoA dehydrogenase, mitochondrial | C134 | ASGAVGLSYGAHSNLcVNQIVR | 1 | -1.2 | -1.3 | 67±5.9 | 51.4±7 |
| Q06BK5 | Ivns1abp | Kelch family protein Nd1-S2 | C214 | SWTScAPLNIR | -1.2 | -1.8 | -1.5 | 41.2±4.3 | 27.6±3 |
| F8VQJ3 | Lamc1 | Laminin subunit gamma-1 | C1598 | TLPTGcFNTPSIEKP | 1 | -1.2 | -1.2 | 67.7±16 | 54.8±10.9 |
| Q9D1L9 | Lamtor5 | Ragulator complex protein LAMTOR5 | C23;C33 | NPSIVGVLcTDSQGLNLGcR | 1.1 | -1.2 | -1.3 | 18.1±1.3 | 13.8±1.7 |
| Q8VEE1 | Lmcd1 | LIM and cysteine-rich domains protein 1 | C243;C246 | EVEYVcELcK | 1.1 | -3.7 | -4 | 16.6±0.9 | 4.2±0.7 |
|  |  |  | C308;C311 | cSGcDEIIFSEDYQR | -1.1 | -2.2 | -2 | 25.2±3.1 | 12.8±2.3 |
| B2RU79 | Ltbp4 | Latent transforming growth factor beta binding protein 4 | C1033;C1045;C1051 | DcDPGYHPGPEGTcDDIDEcR | 1.2 | -2.5 | -3.1 | 82±10.2 | 24.7±2.3 |
| Q922B1 | Macrod1 | O-acetyl-ADP-ribose deacetylase MACROD1 | C197 | AAGSLLTDEcR | -1 | -1.4 | -1.4 | 30.5±5.5 | 21.1±4.5 |
| Q3ULD5 | Mccc2 | Methylcrotonoyl-CoA carboxylase beta chain, mitochondrial | C131 | VSGVEcMIVANDATVK | 1.2 | -1.4 | -1.6 | 34.4±0.6 | 24.5±3 |
|  |  |  | C167 | LPcIYLVDSGGANLPR | 1.3 | -1.4 | -1.6 | 92.4±11.2 | 54.9±8.2 |
| B1ATQ3 | Mdh1 | Malate dehydrogenase 1, NAD (Soluble) (Fragment) | C154 | ENFScLT | 1.1 | -1.2 | -1.3 | 59.2±5.8 | 45.9±5.7 |
| P08249 | Mdh2 | Malate dehydrogenase, mitochondrial | C212 | TIIPLISQcTPK | 1.2 | -1.1 | -1.3 | 176±18.7 | 135.3±13.1 |
|  |  |  | C93 | GcDVVVIPAGVPR | 1 | -1.1 | -1.1 | 175.5±12.6 | 151.4±18.3 |
| G8JL35 | Mob4 | MOB-like protein phocein (Fragment) | C92 | HTLDGAAcLLNSNK | 1.2 | -1.3 | -1.6 | 23.7±3.1 | 15±3.4 |
| Q3TF37 | Mybpc3 | Myosin-binding protein C, cardiac-type | C1040(IAM);C1041 | SIIAGYNAILccAVR | 3 | 1.2 | -2.5 | 816.5±304.2 | 500.6±264 |
| Q9CY45 | N6amt2 | N(6)-adenine-specific DNA methyltransferase 2 | C202;C212 | cYTNYDSGLDcEA | 1.3 | -1 | -1.3 | 18.7±3.1 | 13.5±2.5 |
| E0CZE0 | Nae1 | NEDD8-activating enzyme E1 regulatory subunit | C459 | DDYVHEFcR | 1.1 | -1.2 | -1.3 | 62.2±6.1 | 48.8±6.8 |
| Q9QYG0-2 | Ndrg2 | Isoform 2 of Protein NDRG2 | C64 | ScFQPLFR | 1.1 | -1.6 | -1.7 | 24.6±2.3 | 14.7±2.3 |
|  |  | Isoform 2 of Protein NDRG2 | C241;C260(IAM) | cPVMLVVGDQAPHEDAVVEcNSK | 1 | -1.5 | -1.4 | 73±16.7 | 50.4±10 |
|  |  | NDRG2  (continued) | C241;C260 | cPVMLVVGDQAPHEDAVVEcNSK | 1.1 | -1.4 | -1.4 | 29±2.4 | 20.1±2.8 |
|  |  |  | C241;C260 | cPVmLVVGDQAPHEDAVVEcNSK | 1.1 | -1.3 | -1.4 | 31.6±2.9 | 23.2±3.5 |
|  |  |  | C307 | YFLQGMGYmASScMTR | 1.1 | -1.2 | -1.3 | 12.3±1.4 | 16.1±3.6 |
| Q7TMF3 | Ndufa12 | NADH dehydrogenase [ubiquinone] 1 alpha subcomplex subunit 12 | C92 | WLHcMTDDPPTTNPPTAR | 1.1 | -1.5 | -1.7 | 25.2±4.5 | 14.6±2.9 |
| Q9Z1P6 | Ndufa7 | NADH dehydrogenase [ubiquinone] 1 alpha subcomplex subunit 7 | C55 | LSNNYYcTR | 1.1 | -1.6 | -1.5 | 25.4±3.8 | 15.2±2.7 |
| Q9DC69 | Ndufa9 | NADH dehydrogenase [ubiquinone] 1 alpha subcomplex subunit 9, mitochondrial | C86 | cDVYDIMHLR | 1.1 | -1.2 | -1.3 | 56±6.2 | 42.1±6.4 |
| Q3TCV9 | Npepl1 | Probable aminopeptidase NPEPL1 | C357 | LVLADGVSYAcK | 1.1 | 1 | -1.1 | 22.8±2.3 | 21.9±4.7 |
| Q80X28 | Nrp1 | Nrp1 protein (Fragment) | C54 | cEWLIQAPEPYQR | 1.2 | -1.3 | -1.6 | 39.1±5.4 | 24.4±3.1 |
| Q8C8G9 | Nt5e | 5'-nucleotidase | C355 | TIVYLDGSTQTcR | 1.2 | -1.7 | -2 | 37.6±8.7 | 17.9±2.7 |
| P29758 | Oat | Ornithine aminotransferase, mitochondrial | C150 | VLPMNTGVEAGETAcK | 1.1 | -1.3 | -1.4 | 46.1±3.1 | 31.9±4.4 |
| Q60597-2 | Ogdh | Isoform 2 of 2-oxoglutarate dehydrogenase, mitochondrial | C594 | SMTcPSTGLEEDVLFHIGK | 1 | -2.2 | -2.6 | 5.3±0.7 | 2.6±0.9 |
|  |  |  | C946 | YPNAELAWcQEEHK | 1.1 | -1.5 | -1.7 | 66±7.7 | 40.3±6.4 |
|  |  |  | C477 | VVNAPIFHVNSDDPEAVMYVcK | 1.1 | -1.5 | -1.6 | 60.9±10.2 | 36.1±4.3 |
|  |  |  | C497 | DVVVDLVcYR | 1.1 | -1.4 | -1.5 | 69±13 | 42.9±5.8 |
|  |  |  | C946 | EAQKYPNAELAWcQEEHK | 1.1 | -1.3 | -1.4 | 74.5±7.2 | 52.2±6.9 |
| B2RXT3 | Ogdhl | Ogdhl protein | C942 | YSGAELVWcQEEHK | 1.3 | -1.3 | -1.7 | 61.6±5.5 | 36.8±5.8 |
| Q8VE38-2 | Oxnad1 | Isoform 2 of Oxidoreductase NAD-binding domain-containing protein 1 | C83 | cTLDSEVALR | 1.2 | -1.7 | -2 | 64.4±9.1 | 31.6±1.5 |
| D3Z6F9 | Oxsm | 3-oxoacyl-[acyl-carrier-protein] synthase, mitochondrial (Fragment) | C86 | NIPcSVAAYVPR | 1 | -1.2 | -1.2 | 53.5±4.1 | 46.5±5 |
| Q9WU78-2 | Pdcd6ip | Isoform 2 of Programmed cell death 6-interacting protein | C76 | YYDQIcSIEPK | 1.3 | -1.1 | -1.4 | 37.4±5.9 | 25.9±3.4 |
| P35486 | Pdha1 | Pyruvate dehydrogenase E1 component subunit alpha, somatic form, mitochondrial | C218;C222 | LPcIFIcENNR | 1.2 | -5.5 | -6.8 | 5.3±0.8 | 1±0.1 |
|  |  |  | C181 | NFYGGNGIVGAQVPLGAGIALAcK | 1.1 | -1.3 | -1.3 | 15.6±2.2 | 12.5±3.3 |
| Q8BKZ9 | Pdhx | Pyruvate dehydrogenase protein X component, mitochondrial | C170 | DVSAPPPVSKPPAPTQPSPQPQIPcPAR | 1.1 | -1.3 | -1.4 | 12.4±1.8 | 9.9±2.3 |
| Q8R2U8 | Pdk1 | Pdk1 protein | C219 | HIGSINPNcDVVEVIK | -1 | -1.5 | -1.4 | 20.2±3.6 | 13.6±2.1 |
| O70250 | Pgam2 | Phosphoglycerate mutase 2 | C153 | YAGLKPEELPTcESLK | 1 | -1.2 | -1.2 | 44.5±4.6 | 38.2±7.9 |
|  |  |  | C153 | YAGLKPEELPTcESLKDTIAR | -1 | -1.2 | -1.2 | 52±6.5 | 44.5±8.4 |
| P20918 | Plg | Plasminogen | C245 | NPDGEPRPWcFTTDPTK | 1.1 | -1.7 | -1.9 | 45.5±5.7 | 24.1±3.5 |
|  |  |  | C747;C758;C768 | STELcAGQLAGGVDScQGDSGGPLVcFEK | 1.1 | -1.4 | -1.5 | 76.5±8.5 | 50.4±5.9 |
| E9PZ15 | Pofut1 | GDP-fucose protein O-fucosyltransferase 1 | C158 | IGSDWNAcAmLK | 1.2 | -1.1 | -1.3 | 30.7±4.9 | 23.9±3 |
| A2BGI9 | Ppih | Peptidyl-prolyl cis-trans isomerase | C131 | LPVVISQcGEM | 1.2 | -1.3 | -1.5 | 44.1±3.1 | 30.2±2.7 |
| P63330 | Ppp2ca | Serine/threonine-protein phosphatase 2A catalytic subunit alpha isoform | C266 | NVVTIFSAPNYcYR | 1.1 | -1.8 | -2 | 31±5.9 | 15.4±4.2 |
|  |  |  | C20 | ELDQWIEQLNEcK | 1.1 | -1.4 | -1.5 | 44.3±3.9 | 29.2±6.3 |
| F7BX26 | Ppp5c | Serine/threonine-protein phosphatase (Fragment) | C76 | TEcYGYALGDATR | 1 | -1.7 | -1.7 | 14.5±1.8 | 10.6±5.2 |
| Q9JHL8 | Prdx5 | Peroxiredoxin 5, isoform CRA_a | C96 | GVLFGVPGAFTPGcSK | 1.4 | 1.1 | -1.2 | 93±7 | 71.6±10 |
| D3Z0Y2 | Prdx6 | Peroxiredoxin-6 | C23 | DFTPVcTTELGR | 1.1 | -1.2 | -1.3 | 52.5±2.9 | 40.2±3.4 |
| Q3UM23 | Rnh1 | Ribonuclease inhibitor | C80 | TNELGDGGVGLVLQGLQNPTcK | 1.1 | -1.1 | -1.3 | 6.8±1.7 | 19.1±14.4 |
| E9PYF1 | Rnpep | Aminopeptidase B | C151 | VGEGPGVcWLAPEQTAGK | -1.3 | -1.5 | -1.2 | 31.4±5.9 | 26.9±7.8 |
| Q9Z0U9 | S1pr3 | Sphingosine 1-phosphate receptor 3 | C377 | NRSFQNGVLcK | 1.2 | -1.4 | -1.7 | 18.5±0.3 | 10.9±2.2 |
| Q8K2B3 | Sdha | Succinate dehydrogenase [ubiquinone] flavoprotein subunit, mitochondrial | C654 | TLNEADcATVPPAIR | 1 | -1.3 | -1.2 | 85.7±5.7 | 68.7±7.1 |
|  |  | Succinate dehydrogenase  (continued) | C266 | TYFScTSAHTSTGDGTAMVTR | 1 | -1 | -1.1 | 68.1±7.1 | 60.9±9.2 |
| E9PYY0 | Serpinb6a | Protein Serpinb6a (Fragment) | C102 | TcDLLASFK | 1 | -1.4 | -1.4 | 45.6±5.7 | 32.9±5.5 |
| P32261 | Serpinc1 | Antithrombin-III | C54 | DIPVNPLcIYR | 1.2 | -1.3 | -1.6 | 80.7±4.9 | 50.3±5.7 |
| Q8BJU0-2 | Sgta | Isoform 2 of Small glutamine-rich tetratricopeptide repeat-containing protein alpha | C129 | AIELNPANAVYFcNR | 1.3 | 1.1 | -1.3 | 19.9±0.8 | 15.8±0.4 |
| Q3TFD0 | Shmt2 | Serine hydroxymethyltransferase | C77 | GLELIASENFcSR | 1.8 | 1.2 | -1.4 | 72±7.3 | 47.2±8.7 |
| E9PUV4 | Skp1a | Protein Skp1a (Fragment) | C72 | GLLDVTcK | 1 | -1.6 | -1.6 | 23.7±1.5 | 14.5±2.9 |
| E9PZ58 | Sms | Spermine synthase | C237 | TcGDVLDNLR | 1.1 | -1.5 | -1.6 | 29.1±3.9 | 18.4±2.7 |
| P08228 | Sod1 | Superoxide dismutase [Cu-Zn] | C147 | LAcGVIGIAQ | 1.1 | -1.2 | -1.3 | 43.9±4.4 | 34.4±6.8 |
| Q91XH5 | Spr | Sepiapterin reductase | C262 | DTFQSGAHVDFYDc | 1.1 | -5.1 | -4.9 | 25.6±5.9 | 4.7±0.8 |
| Q3UDS4 | Sqrdl | Sulfide:quinone oxidoreductase, mitochondrial | C127 | VAELNPDENcIR | 1.4 | -2.4 | -3.3 | 12.8±4.1 | 3.9±1.1 |
| Q9Z2I9 | Sucla2 | Succinyl-CoA ligase [ADP-forming] subunit beta, mitochondrial | C430 | ILAcDDLDEAAK | 1.2 | -3.2 | -3.4 | 6.5±0.6 | 3.8±1.3 |
| Q3U630 | Tars | Putative uncharacterized protein | C106 | TTPYQIAcGISQGLADNTVVAK | 1.1 | -1.3 | -1.2 | 44.1±16.3 | 33.8±6.4 |
| O88968 | Tcn2 | Transcobalamin-2 | C312 | TYLDLIFPDcQASR | 1.2 | -1.2 | -1.3 | 74.2±12.5 | 38.5±11.9 |
| Q921I1 | Tf | Serotransferrin | C246 | DQYELLcLDNTR | 1.3 | -1.2 | -1.5 | 40.2±6.1 | 25.6±3.5 |
| P21981 | Tgm2 | Protein-glutamine gamma-glutamyltransferase 2 | C669 | LVVNFQcDK | 1.3 | -1.5 | -1.9 | 25.1±11.1 | 13.9±7.5 |
| Q9WVA2 | Timm8a1 | Mitochondrial import inner membrane translocase subunit Tim8 A | C43 | FQQLVHQMTELcWEK | 1.1 | -1.1 | -1.2 | 42.5±6.6 | 32.8±3.3 |
| P26039 | Tln1 | Talin-1 | C2442 | QVAASTAQLLVAcK | 1.2 | -1.2 | -1.5 | 14.8±3.3 | 10±2.8 |
| Q922K4 | Tpp2 | Tpp2 protein (Fragment) | C195 | STLIDALcR | 1.2 | -1.1 | -1.2 | 18.3±1.9 | 14.9±3 |
| Q9D8Z2 | Triap1 | TP53-regulated inhibitor of apoptosis 1 | C37 | GDGSGDPcTDLFK | -1 | -1.3 | -1.2 | 39.4±3.2 | 32±5.8 |
| A2ASS6-2 | Ttn | Isoform 2 of Titin | C23572 | VLAcNAGGPGEPAEVPGTVK | 1.7 | -1.3 | -2.3 | 46.2±4.3 | 20.4±1.1 |
| Q8VBT1 | Txlnb | Beta-taxilin | C435 | EYEcFVMK | 1.2 | -1.3 | -1.5 | 21.7±1 | 14.8±1.7 |
| Q02053 | Uba1 | Ubiquitin-like modifier-activating enzyme 1 | C234 | DNPGVVTcLDEAR | 1.1 | -1.3 | -1.4 | 126.7±6.2 | 94.5±13.6 |
| P61089 | Ube2n | Ubiquitin-conjugating enzyme E2 N | C87 | IcLDILK | 1.2 | -1.2 | -1.5 | 18.6±6.4 | 12.8±5.8 |
| Q9JKB1 | Uchl3 | Ubiquitin carboxyl-terminal hydrolase isozyme L3 | C95 | QTISNAcGTIGLIHAIANNK | 1.2 | -1.5 | -1.7 | 22.5±10.2 | 13.6±7.1 |
| P70697 | Urod | Uroporphyrinogen decarboxylase | C35 | AAWGEETDYTPVWcMR | -1.3 | -1.3 | 1.1 | 17.6±8.4 | 20±11.6 |
| Q3U6K8 | Vdac1 | Voltage-dependent anion-selective channel protein 1 | C232 | YQVDPDAcFSAK | 1.1 | -2 | -2.1 | 8.1±2.4 | 7.3±2.3 |
| G3UX26 | Vdac2 | Voltage-dependent anion-selective channel protein 2 (Fragment) | C199;C216 | VcEDFDTSVNLAWTSGTNcTR | 1 | -24.4 | -23.6 | 9.7±3.2 | 0.6±0.2 |
|  |  |  | C65 | WcEYGLTFTEK | 1.1 | -2.7 | -2.8 | 9.3±1 | 4.9±2 |
|  |  |  | C36 | ScSGVEFSTSGSSNTDTGK | 1.2 | -2.4 | -2 | 6.1±1.7 | 5.3±0.3 |
|  |  |  | C65 | YKWcEYGLTFTEK | -1.3 | -2.4 | -1.8 | 25.6±9.5 | 13±2.7 |
| Q99KC8 | Vwa5a | von Willebrand factor A domain-containing protein 5A | C374 | ANLGGTEILTPLcNIYK | 1.1 | -1.4 | -1.4 | 17.7±0.8 | 12.1±2.5 |
