# Supplementary material for: Overexpression of Catalase Diminishes Oxidative Cysteine Modifications of Cardiac Proteins
Source: PLoS One. 2015 Dec 7;10(12):e0144025. doi: 10.1371/journal.pone.0144025 (PMC4671598; doi:10.1371/journal.pone.0144025)
Supplement: S2 Table — Accession number, gene ID, sites of modification and peptide sequences were retrieved from the Uniprot knowledgebase. Fold changes in Cat Tg vs. WT, were calculated from ratio of reporter ions for changes in total available cysteine as (m/z 129)/(m/z 127), reversibly oxidized cysteine thiols as (m/z 128)/(m/z 126) and the thiol occupancy as ((m/z 128)/ (m/z 126))/((m/z 129)/(m/z 127)). The thiol occupancy columns indicate percentage thiol occupancy, calculated as (m/z 126)/(m/z 127) for WT, and (m/z 128) /(m/z 129) for Cat Tg, together with The standard error mean (SEM) was calculated from N = 5 biological replicates. (DOCX) [file pone.0144025.s006.docx]

| **Accession No.** | **GN** | **Protein description** | **Cys sites** | **Sequence** | **Fold changes (Cat Tg vs WT)** | | | **Occupancy± SEM (%)** | |
| --- | --- | --- | --- | --- | --- | --- | --- | --- | --- |
|  |  |  |  |  |  |  |  |  |  |
|  |  |  |  |  | **Total available Cys** | **Reversibly oxidized Cys** | **Cys thiol occupancy** | **WT** | **Cat Tg** |
| Q3UPU8 | Acaa1a | 3-ketoacyl-CoA thiolase A, peroxisomal | C177 | DcLTPMGMTSENVAER | 1.5 | -1.5 | -2.1 | 36.8±3.9 | 17.9±1.9 |
| Q9D7B6 | Acad8 | Isobutyryl-CoA dehydrogenase, mitochondrial | C157 | FASYcLTEPGSGSDAASLLTSAK | 1.1 | -2.6 | -3.2 | 10.1±2.1 | 2.5±0.6 |
| E9Q705 | Bola3 | BolA-like protein 3 | C47 | ATAIQVTDISGGcGAMYEIK | 1 | -2.9 | -2.9 | 10.6±1 | 3.8±0.6 |
| Q3UMI9 | Cand1 | Cullin-associated NEDD8-dissociated protein 1 | C237 | TYIQcIAAISR | 1.1 | -2.2 | -2.3 | 19.7±1.2 | 8.7±1.4 |
| Q3UF58 | Cat | Catalase | C376 | LGPNYLQIPVNcPYR | 14 | -1.7 | -24.5 | 35.5±3.8 | 1.9±0.3 |
| P48758 | Cbr1 | Carbonyl reductase [NADPH] 1 | C226;C227 | ILLNAccPGWVR | 1.4 | -2.1 | -3 | 17.9±5.3 | 6.2±2.7 |
| Q8R4N0 | Clybl | Citrate lyase subunit beta-like protein, mitochondrial | C67;C72 | VDcAVLDcEDGVAENK | 1.1 | -2 | -2.1 | 46.3±7.6 | 19.8±2.9 |
| P97315 | Csrp1 | Cysteine and glycine-rich protein 1 | C58 | NLDSTTVAVHGEEIYcK | 1 | -4.1 | -4.2 | 24±2.8 | 5.7±1 |
| P50462 | Csrp3 | Cysteine and glycine-rich protein 3 | C79 | GIGFGQGAGcLSTDTGEHLGLQFQQSPKPAR | -1 | -3.1 | -3 | 6.6±0.8 | 4.4±1.4 |
| Q3TQ74 | Ehd4 | EH domain-containing protein 4 | C141 | FMcSQLPNQVLK | 1.3 | -1.7 | -2.2 | 17.8±1.9 | 8.4±1.4 |
| D3Z1B2 | Gm4953 | MCG50540 | C101 | ncAEFVSGSQLR | 1.5 | -2.2 | -3.2 | 32.1±8.1 | 9±1.7 |
| Q61642 | H2-K1 | H-2K-sm1 | C357 | GGDYALAPGSQTSDLSLPDcK | 1.4 | -1.8 | -2.3 | 42.3±4.3 | 17.7±5.4 |
| Q8BIJ6 | Iars2 | Isoleucine--tRNA ligase, mitochondrial | C465 | EENIVHSYPcDWR | 1.1 | -2.1 | -2.4 | 15.4±1.7 | 6.5±0.9 |
| Q9D6R2-2 | Idh3a | Isoform 2 of Isocitrate dehydrogenase [NAD] subunit alpha, mitochondrial | C273;C281 | cSDFTEEIcR | 1.1 | -3.6 | -4 | 6±0.7 | 1.7±0.2 |
|  |  |  | C49 | TFDLYANVRPcVSIEGYK | 1.1 | -1.9 | -2.1 | 14.3±0.9 | 7±1.1 |
| Q8VEE1 | Lmcd1 | LIM and cysteine-rich domains protein 1 | C243;C246 | EVEYVcELcK | 1.1 | -3.7 | -4 | 16.6±0.9 | 4.2±0.7 |
| B2RU79 | Ltbp4 | Latent transforming growth factor beta binding protein 4 | C1033;C1045;C1051 | DcDPGYHPGPEGTcDDIDEcR | 1.2 | -2.5 | -3.1 | 82±10.2 | 24.7±2.3 |
| Q8C8G9 | Nt5e | 5'-nucleotidase | C355 | TIVYLDGSTQTcR | 1.2 | -1.7 | -2 | 37.6±8.7 | 17.9±2.7 |
| Q60597-2 | Ogdh | Isoform 2 of 2-oxoglutarate dehydrogenase, mitochondrial | C594 | SMTcPSTGLEEDVLFHIGK | 1 | -2.2 | -2.6 | 5.3±0.7 | 2.6±0.9 |
| P35486 | Pdha1 | Pyruvate dehydrogenase E1 component subunit alpha, somatic form, mitochondrial | C218;C222 | LPcIFIcENNR | 1.2 | -5.5 | -6.8 | 5.3±0.8 | 1±0.1 |
| Q91XH5 | Spr | Sepiapterin reductase | C262 | DTFQSGAHVDFYDc | 1.1 | -5.1 | -4.9 | 25.6±5.9 | 4.7±0.8 |
| Q3UDS4 | Sqrdl | Sulfide:quinone oxidoreductase, mitochondrial | C127 | VAELNPDENcIR | 1.4 | -2.4 | -3.3 | 12.8±4.1 | 3.9±1.1 |
| Q9Z2I9 | Sucla2 | Succinyl-CoA ligase [ADP-forming] subunit beta, mitochondrial | C430 | ILAcDDLDEAAK | 1.2 | -3.2 | -3.4 | 6.5±0.6 | 3.8±1.3 |
| Q3U6K8 | Vdac1 | Voltage-dependent anion-selective channel protein 1 | C232 | YQVDPDAcFSAK | 1.1 | -2 | -2.1 | 8.1±2.4 | 7.3±2.3 |
| G3UX26 | Vdac2 | Voltage-dependent anion-selective channel protein 2 (Fragment) | C199;C216 | VcEDFDTSVNLAWTSGTNcTR | 1 | -24.4 | -23.6 | 9.7±3.2 | 0.6±0.2 |
|  |  |  | C65 | WcEYGLTFTEK | 1.1 | -2.7 | -2.8 | 9.3±1 | 4.9±2 |
|  |  |  | C36 | ScSGVEFSTSGSSNTDTGK | 1.2 | -2.4 | -2 | 6.1±1.7 | 5.3±0.3 |
