# Supplementary material for: Overexpression of Catalase Diminishes Oxidative Cysteine Modifications of Cardiac Proteins
Source: PLoS One. 2015 Dec 7;10(12):e0144025. doi: 10.1371/journal.pone.0144025 (PMC4671598; doi:10.1371/journal.pone.0144025)
Supplement: S3 Table — Accession number, gene ID, sites of modification and peptide sequences were retrieved from the Uniprot knowledgebase. Fold changes in Cat Tg vs. WT, were calculated from ratio of reporter ions for changes in total available cysteine as (m/z 129)/(m/z 127), reversibly oxidized cysteine thiols as (m/z 128)/(m/z 126) and the thiol occupancy as ((m/z 128)/ (m/z 126))/((m/z 129)/(m/z 127)). The thiol occupancy columns indicate percentage thiol occupancy, calculated as (m/z 126)/(m/z 127) for WT, and (m/z 128) /(m/z 129) for Cat Tg, together with The standard error mean (SEM) was calculated from N = 5 biological replicates. (DOCX) [file pone.0144025.s007.docx]

| **Accession No.** | **GN** | **Protein description** | **Cys sites** | **Sequence** | **Fold changes (Cat Tg vs WT)** | | | **Occupancy± SEM (%)** | |
| --- | --- | --- | --- | --- | --- | --- | --- | --- | --- |
|  |  |  |  |  |  |  |  |  |  |
|  |  |  |  |  | **Total available Cys** | **Reversibly oxidized Cys** | **Cys thiol occupancy** | **WT** | **Cat Tg** |
| **1. Mitochondrial dysfunction** | | |  |  |  |  |  |  |  |
| Q99KI0 | Aco2 | Aconitate hydratase, mitochondrial | C385 | VGLIGScTNSSYEDmGR | 1.1 | -1.4 | -1.4 | 43.4±6.1 | 28.9±5 |
| Q3UF58 | Cat | Catalase | C376 | LGPNYLQIPVNcPYR | 14 | -1.7 | -24.5 | 35.5±3.8 | 1.9±0.3 |
| O08756 | Hsd17b10 | 3-hydroxyacyl-CoA dehydrogenase type-2 | C58 | LGEScIFAPANVTSEK | 1.1 | -1.6 | -1.6 | 16±3.6 | 10.7±3.9 |
| Q7TMF3 | Ndufa12 | NADH dehydrogenase [ubiquinone] 1 alpha subcomplex subunit 12 | C92 | WLHcMTDDPPTTNPPTAR | 1.1 | -1.5 | -1.7 | 25.2±4.5 | 14.6±2.9 |
| Q9Z1P6 | Ndufa7 | NADH dehydrogenase [ubiquinone] 1 alpha subcomplex subunit 7 | C55 | LSNNYYcTR | 1.1 | -1.6 | -1.5 | 25.4±3.8 | 15.2±2.7 |
| Q60597-2 | Ogdh | Isoform 2 of 2-oxoglutarate dehydrogenase, mitochondrial | C594 | SMTcPSTGLEEDVLFHIGK | 1 | -2.2 | -2.6 | 5.3±0.7 | 2.6±0.9 |
|  |  |  | C946 | YPNAELAWcQEEHK | 1.1 | -1.5 | -1.7 | 66±7.7 | 40.3±6.4 |
|  |  |  | C477 | VVNAPIFHVNSDDPEAVMYVcK | 1.1 | -1.5 | -1.6 | 60.9±10.2 | 36.1±4.3 |
|  |  |  | C497 | DVVVDLVcYR | 1.1 | -1.4 | -1.5 | 69±13 | 42.9±5.8 |
|  |  |  | C946 | EAQKYPNAELAWcQEEHK | 1.1 | -1.3 | -1.4 | 74.5±7.2 | 52.2±6.9 |
| P35486 | Pdha1 | Pyruvate dehydrogenase E1 component subunit alpha, somatic form, mitochondrial | C218;C222 | LPcIFIcENNR | 1.2 | -5.5 | -6.8 | 5.3±0.8 | 1±0.1 |
| Q3U6K8 | Vdac1 | Voltage-dependent anion-selective channel protein 1 | C232 | YQVDPDAcFSAK | 1.1 | -2 | -2.1 | 8.1±2.4 | 7.3±2.3 |
|  |  |  | C199;C216 | VcEDFDTSVNLAWTSGTNcTR | 1 | -24.4 | -23.6 | 9.7±3.2 | 0.6±0.2 |
| G3UX26 | Vdac2 | Voltage-dependent anion-selective channel protein 2 (Fragment) | C65 | WcEYGLTFTEK | 1.1 | -2.7 | -2.8 | 9.3±1 | 4.9±2 |
|  |  |  | C36 | ScSGVEFSTSGSSNTDTGK | 1.2 | -2.4 | -2 | 6.1±1.7 | 5.3±0.3 |
|  |  |  | C65 | YKWcEYGLTFTEK | -1.3 | -2.4 | -1.8 | 25.6±9.5 | 13±2.7 |
| **2. Acute phase response signaling** | | |  |  |  |  |  |  |  |
| P29699 | Ahsg | Alpha-2-HS-glycoprotein | C114 | QLTEHAVEGDcDFHILK | 1.2 | -1.5 | -1.8 | 56.3±3.5 | 31.4±4.1 |
| P07724 | Alb | Serum albumin | C591 | DTcFSTEGPNLVTR | 1.1 | -1.8 | -1.8 | 92.5±18.4 | 43.3±5.8 |
|  |  |  | C269;C270;C277 | VNKEccHGDLLEcADDRAELAK | 1.1 | -1.4 | -1.5 | 30.7±1.2 | 20.4±1.9 |
|  |  |  | C269;C270;C277 | EccHGDLLEcADDRAELAK | 1.1 | -1.3 | -1.5 | 35.1±4.4 | 23.5±1.8 |
|  |  |  | C269;C270;C277 | VNKEccHGDLLEcADDR | 1.1 | -1.2 | -1.4 | 31.6±3.5 | 22.4±2.2 |
| Q07456 | Ambp | Protein AMBP | C336 | EYcGVPGDGYEELIR | 1.1 | -1.4 | -1.6 | 62.7±3.6 | 42.6±6.1 |
| P01027 | C3 | Complement C3 | C1513;C1518 | cAEENcFMQQSQEK | 1.2 | -1.5 | -1.8 | 52.1±16.7 | 29.6±10.8 |
| B8JJM3 | Cfb | Complement factor B (Fragment) | C176;C189 | FLcTGGVDPYADPNTcK | 1.2 | -1.4 | -1.6 | 79±2.5 | 45.6±4.7 |
| H7BX99 | F2 | Prothrombin | C214 | DNLSPPLGQcLTER | 1.2 | -1.6 | -1.8 | 56.1±4.1 | 32.8±5.7 |
|  |  |  | C546 | ITDNMFcAGFK | 1 | -1.5 | -1.6 | 45.5±5.1 | 29.3±5.1 |
| P20918 | Plg | Plasminogen | C245 | NPDGEPRPWcFTTDPTK | 1.1 | -1.7 | -1.9 | 45.5±5.7 | 24.1±3.5 |
|  |  |  | C747;C758;C768 | STELcAGQLAGGVDScQGDSGGPLVcFEK | 1.1 | -1.4 | -1.5 | 76.5±8.5 | 50.4±5.9 |
| **3. TCA cycle II** | |  |  |  |  |  |  |  |  |
| Q99KI0 | Aco2 | Aconitate hydratase, mitochondrial | C385 | VGLIGScTNSSYEDmGR | 1.1 | -1.4 | -1.4 | 43.4±6.1 | 28.9±5 |
| Q9D6R2-2 | Idh3a | Isoform 2 of Isocitrate dehydrogenase [NAD] subunit alpha, mitochondrial | C273;C281 | cSDFTEEIcR | 1.1 | -3.6 | -4 | 6±0.7 | 1.7±0.2 |
|  |  |  | C49 | TFDLYANVRPcVSIEGYK | 1.1 | -1.9 | -2.1 | 14.3±0.9 | 7±1.1 |
| Q684I8 | Idh3g | Isocitrate dehydrogenase 3 (NAD+), gamma (Fragment) | C146 | TSLDLYANVIHcK | 1.2 | -1.5 | -1.8 | 20.8±2.6 | 11.8±1.4 |
|  |  |  | C233;C234 | LGDGLFLQccR | 1.1 | -1.6 | -1.7 | 20±2.6 | 11.7±1.8 |
|  |  |  | C79 | HAcVPVDFEEVHVSSnADEEDIR | 1.3 | -1.3 | -1.6 | 23.6±2.8 | 14.4±0.4 |
| Q60597-2 | Ogdh | Isoform 2 of 2-oxoglutarate dehydrogenase, mitochondrial | C594 | SMTcPSTGLEEDVLFHIGK | 1 | -2.2 | -2.6 | 5.3±0.7 | 2.6±0.9 |
|  |  |  | C946 | YPNAELAWcQEEHK | 1.1 | -1.5 | -1.7 | 66±7.7 | 40.3±6.4 |
|  |  |  | C477 | VVNAPIFHVNSDDPEAVMYVcK | 1.1 | -1.5 | -1.6 | 60.9±10.2 | 36.1±4.3 |
|  |  |  | C497 | DVVVDLVcYR | 1.1 | -1.4 | -1.5 | 69±13 | 42.9±5.8 |
|  |  |  | C946 | EAQKYPNAELAWcQEEHK | 1.1 | -1.3 | -1.4 | 74.5±7.2 | 52.2±6.9 |
| Q9Z2I9 | Sucla2 | Succinyl-CoA ligase [ADP-forming] subunit beta, mitochondrial | C430 | ILAcDDLDEAAK | 1.2 | -3.2 | -3.4 | 6.5±0.6 | 3.8±1.3 |
| **4. NRF2-mediated oxidative stress** | | |  |  |  |  |  |  |  |
| Q3UF58 | Cat | Catalase | C376 | LGPNYLQIPVNcPYR | 14 | -1.7 | -24.5 | 35.5±3.8 | 1.9±0.3 |
| P48758 | Cbr1 | Carbonyl reductase [NADPH] 1 | C226;C227 | ILLNAccPGWVR | 1.4 | -2.1 | -3 | 17.9±5.3 | 6.2±2.7 |
| Q3UDB1 | Cct7 | Putative uncharacterized protein (Fragment) | C450 | QLcDNAGFDATNILNK | 1.2 | -1.4 | -1.8 | 37.1±3.9 | 21±4.3 |
| P10649 | Gstm1 | Glutathione S-transferase Mu 1 | C115 | mQLIMLcYNPDFEK | 1.1 | -1.4 | -1.5 | 28.2±1.4 | 19.1±2 |
| E9PVM7 | Gstm5 | Glutathione S-transferase Mu 5 (Fragment) | C177 | cLDEFPNLK | 1.1 | -1.3 | -1.4 | 44.9±3.2 | 33.1±4.5 |
| **5. Fatty Acid β-oxidation I** | | |  |  |  |  |  |  |  |
| Q3UPU8 | Acaa1a | 3-ketoacyl-CoA thiolase A, peroxisomal | C177 | DcLTPMGMTSENVAER | 1.5 | -1.5 | -2.1 | 36.8±3.9 | 17.9±1.9 |
| D3Z041 | Acsl1 | Long-chain-fatty-acid--CoA ligase 1 | C55 | ALKPPcDLSMQSVEIAGTTDGIR | 1.1 | -1.2 | -1.5 | 11.5±2.4 | 6.4±1.2 |
|  |  |  | C626 | GLQGSFEELcR | 1.2 | -1.3 | -1.5 | 24.3±2.8 | 16.5±3.2 |
|  |  |  | C275 | VKPKPPEPEDLAIIcFTSGTTGNPK | 1.2 | -1.2 | -1.5 | 36.9±6.3 | 24.9±4.8 |
|  |  |  | C298 | GAmITHQNIINDcSGFIK | 1.2 | -1.3 | -1.5 | 39.9±6.5 | 26±3.8 |
|  |  |  | C109 | GIQVSNNGPcLGSR | 1.2 | -1.2 | -1.4 | 46.7±8 | 34.1±5.7 |
|  |  |  | C298 | GAMITHQNIINDcSGFIK | 1.2 | -1.2 | -1.3 | 51.4±9.1 | 36.3±5.7 |
| O08756 | Hsd17b10 | 3-hydroxyacyl-CoA dehydrogenase type-2 | C58 | LGEScIFAPANVTSEK | 1.1 | -1.6 | -1.6 | 16±3.6 | 10.7±3.9 |
